# PtdIns4P on Dispersed Trans-Golgi Network Mediates NLRP3 Inflammasome Activation

**DOI:** 10.1038/s41586-018-0761-3

**Published:** 2018-11-28

**Authors:** Jueqi Chen, Zhijian J. Chen

**Affiliations:** 1 Department of Molecular Biology, University of Texas Southwestern Medical Center, Dallas, TX 75390-9148; 2 Howard Hughes Medical Institute, University of Texas Southwestern Medical Center, Dallas, TX 75390-9148

## Abstract

The NLRP3 inflammasome, which has been linked to human inflammatory diseases, is activated by a plethora of stimuli. How NLRP3 is activated by such diverse stimuli is a central question that is unresolved. Here we show that different NLRP3 stimuli lead to a hitherto unknown disassembly of trans-Golgi network (TGN). NLRP3 is recruited to the dispersed TGN (dTGN) through ionic bonding between a conserved polybasic region in NLRP3 and the negatively-charged phosphatidylinositol 4-phosphate (PI4P) on dTGN. dTGN then serves as a scaffold for NLRP3 aggregation into multiple puncta, which polymerize the adaptor ASC to activate the downstream signaling cascade. Disruption of interaction between NLRP3 and PI4P on dTGN blocked NLRP3 aggregation and signaling. These results indicate that recruitment of NLRP3 to dTGN is an early and common cellular event that leads to NLRP3 aggregation and activation in response to diverse stimuli.

Inflammasomes are multi-protein complexes that serve as a platform for caspase-1-depedent activation of proinflammatory cytokines including interleukin-1β (IL-1β), as well as induction of a specific form of inflammatory cell death termed pyroptosis. Among them, the NLRP3 inflammasome is unique in that it can be triggered by a large array of stimuli such as nigericin (an antibiotic from *Streptomyces hygroscopicus*) and ATP released from damaged cells^1^. Deregulated NLRP3 stimulation can lead to uncontrolled infection, autoimmune diseases, neurodegenerative diseases, metabolic disorders, and many other human illnesses^2^. Given the chemical and structural diversity of these stimuli, and the lack of evidence that NLRP3 directly interacts with any of these molecules, the mechanism by which NLRP3 is activated has remained an enigma. After stimulation, NLRP3 recruits the adaptor protein ASC (also known as PYCARD)^3^. ASC then undergoes prion-like polymerization and forms a single large spherical structure (“speck”) in the perinuclear region^4,5^, before recruiting caspase-1 to activate the downstream cascade^1^.

Through a combination of biochemical, imaging and genetic approaches in both reconstituted systems and primary macrophages, we have discovered a new common cellular signal downstream of diverse NLRP3 stimuli: the disassembly of trans-Golgi network (TGN) into various dispersed structures (dTGN). NLRP3 is then recruited to dTGN through an interaction between a polybasic region on NLRP3 and the negatively-charged phosphatidylinositol 4-phosphate (PI4P) on dTGN. NLRP3 on dTGN forms multiple puncta that induce ASC polymerization, thereby activating the downstream signaling cascade.

## An in vitro assay for detection of NLRP3 activity

**Extended Data Figure 1.**
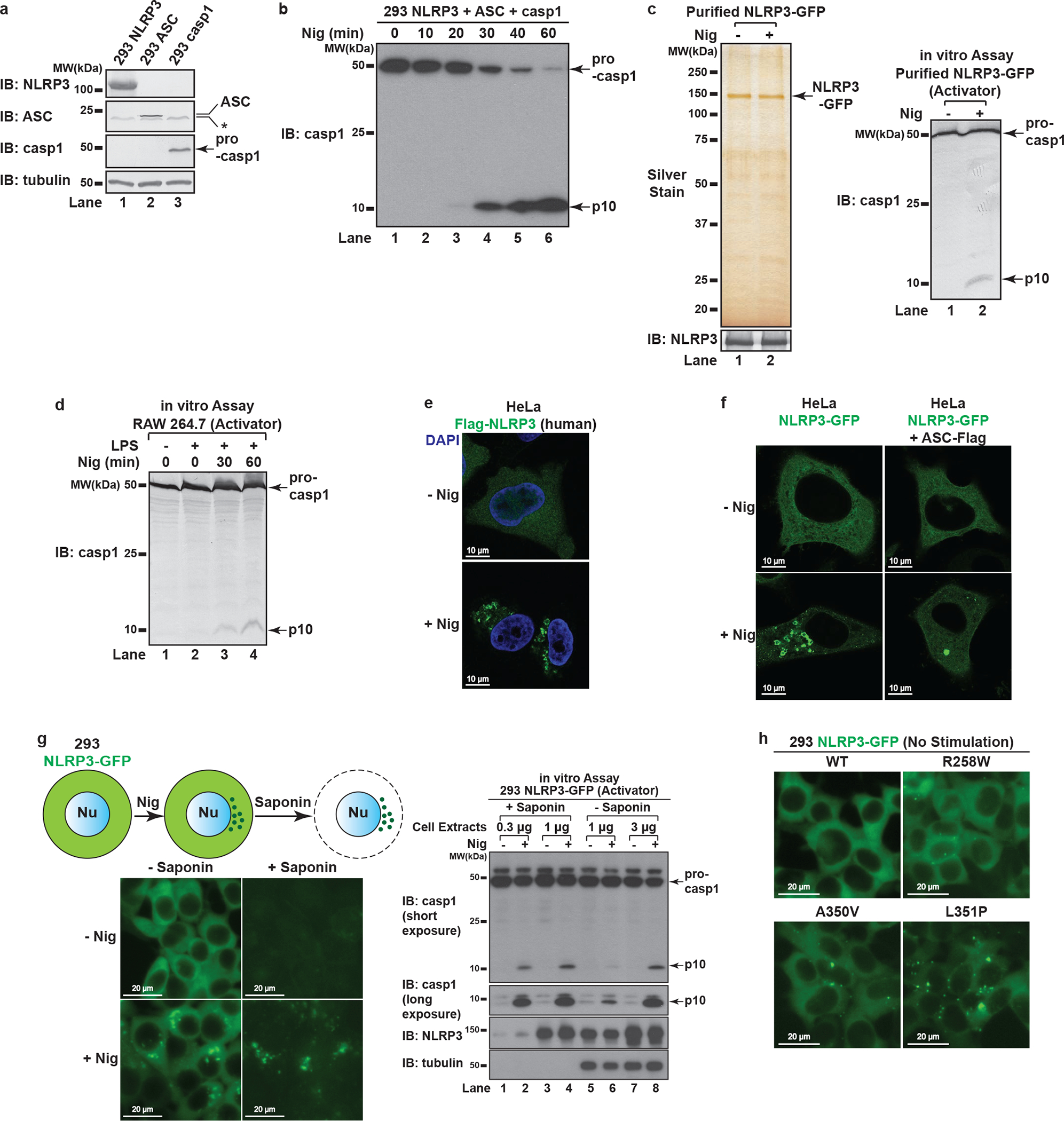
NLRP3 forms multiple puncta to activate the inflammasome pathway. **a**, Endogenous NLRP3, ASC and caspase-1 are not detectable in HEK293T. Extracts from HEK293T cell lines stably expressing the indicated proteins were examined by immunoblotting. *, a nonspecific band. **b**, Reconstitution of NLRP3 inflammasome pathway in HEK293T. Cells stably expressing murine NLRP3, ASC and caspase-1 were treated with nigericin (Nig) (10 μM) for 60 minutes followed by immunoblotting. **c**, Highly purified NLRP3 showed signal-dependent activity in the in vitro assay. NLRP3-GFP was purified through fractionation and immunoprecipitation as detailed in Methods, before the purity and activity were examined by silver staining (left panel) and the in vitro assay (right panel) respectively. **d**, The in vitro assay detects activation of endogenous NLRP3. RAW 264.7 was treated with LPS (50 ng/mL) for 3 hours and stimulated with nigericin (10 μM) for 60 minutes before cell extracts were collected for the in vitro NLRP3 activity assay. **e**, Human NLRP3 also formed various puncta in response to nigericin. HeLa cells stably expressing human Flag-NLRP3 were treated with nigericin (10 μM) for 80 minutes before immunostaining with a Flag antibody. **f**, NLRP3 formed multiple puncta in the absence of ASC but a large speck in the presence of ASC. Cells stably expressing the indicated proteins were treated as in (**e**) before imaging. **g**, NLRP3 puncta possessed high activity. Left panel: nigericin (10 μM, 60 minutes)-induced NLRP3 puncta remained in the cells after saponin treatment. Nu, Nucleus. Right panel: cell extracts with or without saponin treatment were examined by the in vitro assay. The p10 level in Lane 4 is approximately 6.5 fold of that in Lane 6 based on quantification in imageJ (normalization by NLRP3 bands intensity). Only Activator cell extracts were used for tubulin immunoblot. **h**, Constitutively active mutants of NLRP3 formed puncta without stimulation. Cells stably expressing the indicated proteins were imaged in the absence of stimulation.

**Figure 1.**
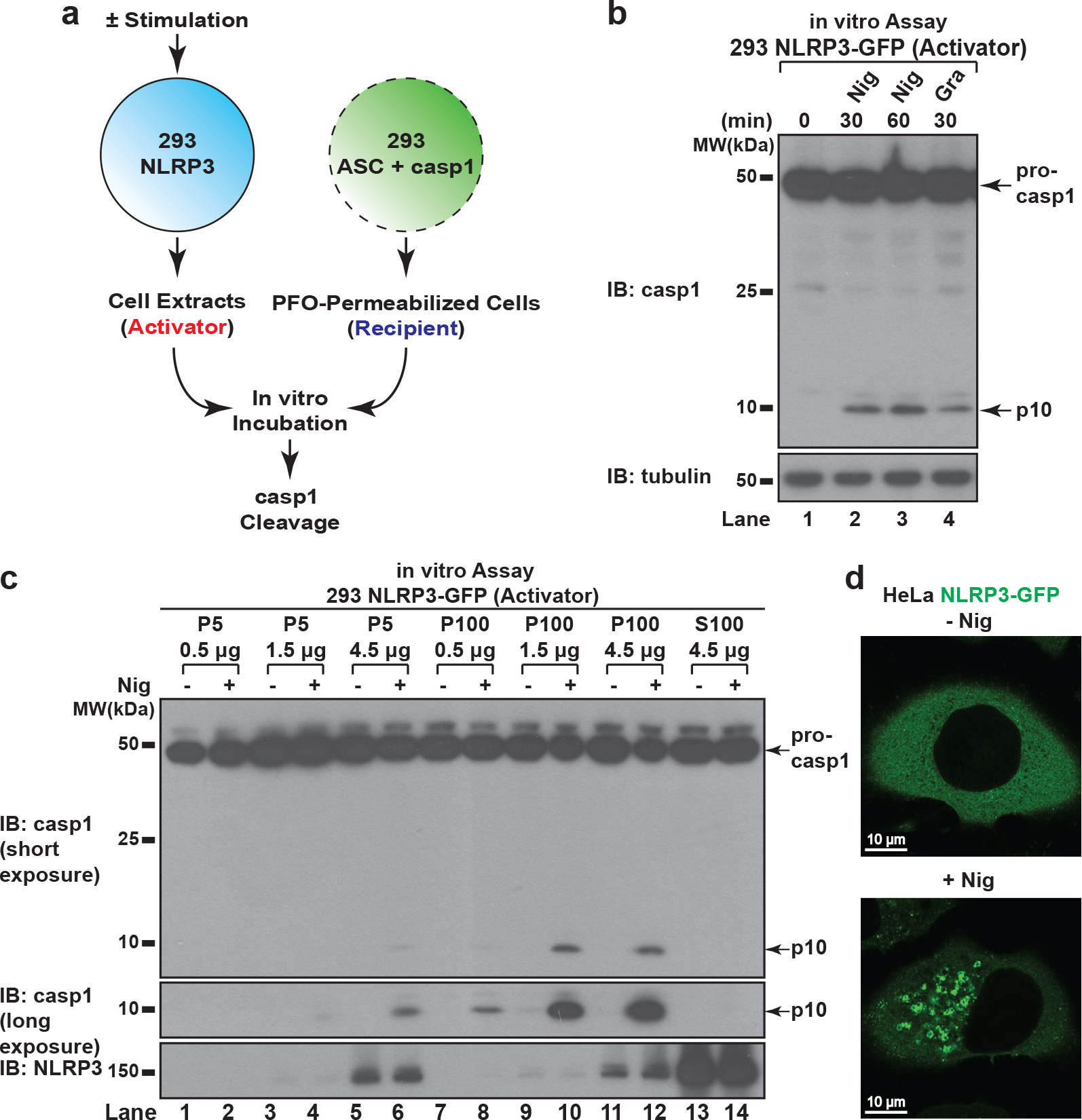
NLRP3 forms multiple puncta after stimulation. **a**, **b**, In vitro assay for examining NLRP3 activity. 293 NLRP3 cells were stimulated with either nigericin (Nig) (10 μM) or gramicidin (Gra) (5 μM) for 60 minutes, before cell extracts were collected and mixed with PFO-permeabilized 293 ASC + casp1 cells. After incubation, the reaction mix was analyzed by immunoblotting. **c**, NLRP3 activity resided in light membrane (P100) and to a less extent heavy membrane (P5), but not in cytosol (S100). The fractions served as “Activator” as in (**a**) in the NLRP3 activity assay. **d**, HeLa cells stably expressing NLRP3-GFP were stimulated −/+ nigericin (10 μM) for 80 minutes before imaging.

We reconstituted NLRP3 pathway in the HEK293T cell line that does not express endogenous NLRP3, ASC or caspase-1 (Extended Data Fig. 1a–b). With this system, we established a biochemical assay aiming at examining specifically the step of NLRP3 activation (Fig. 1a). Basically, we set up HEK293T cell lines stably expressing either NLRP3 (293 NLRP3) or ASC and caspase-1 (293 ASC + casp1). After stimulation of 293 NLRP3 (“Activator”), its cell extracts were mixed with 293 ASC + casp1 (“Recipient”) permeabilized with perfringolysin O (PFO), a bacterial toxin that pokes holes on plasma membrane^6^. After incubation, caspase-1 cleavage was detected in a manner dependent on treatment of NLRP3 stimuli such as nigericin and gramicidin (Fig. 1b). Highly purified NLRP3 also had activity in this assay in a stimulus-dependent manner (Extended Data Fig. 1c), suggesting that the activity came from NLRP3 itself. The Activator cell could also be replaced with RAW 264.7, a macrophage-like cell line that expresses endogenous NLRP3 but not ASC^7^ (Extended Data Fig. 1d). Thus, the in vitro assay faithfully recapitulates the activation status of both exogenous and endogenous NLRP3.

With this in vitro assay, we tested in which subcellular fraction NLRP3 activity resides. Cell extracts from 293 NLRP3-GFP were fractionated into P5 (heavy membrane), P100 (light membrane) and S100 (cytosol) fractions through differential centrifugation, followed by NLRP3 activity assay for each fraction. Even though the majority of NLRP3 was in S100 (cytosol), NLRP3 activity was only detected in P100 (light membrane) and to a lesser extent P5 (heavy membrane) (Fig. 1c), indicating that only a small fraction of NLRP3 becomes active upon stimulation, and it is associated with membranes and/or forms large aggregates.

## NLRP3 aggregates on dispersed TGN

Fluorscent microscopy experiments showed that NLRP3-GFP was diffused across the cytosol under basal conditions but formed multiple small puncta upon nigericin treatment (Fig. 1d and Extended Data Fig. 1e). These puncta were dramatically different from the single large speck formed when ASC was present (Extended Data Fig. 1f), suggesting that NLRP3 first aggregates into multiple small puncta before being incorporated into a large speck with ASC. Enrichment of NLRP3 puncta through saponin treatment showed that these puncta had higher specific activity than NLRP3 in crude cell extracts (Extended Data Fig. 1g), indicating that these puncta are the active form of NLRP3. Interestingly, the constitutively active disease mutants^8,9^ of NLRP3 could form multiple puncta without any stimulation (Extended Data Fig. 1h), again suggesting that NLRP3 requires aggregation to become active.

**Extended Data Figure 2.**
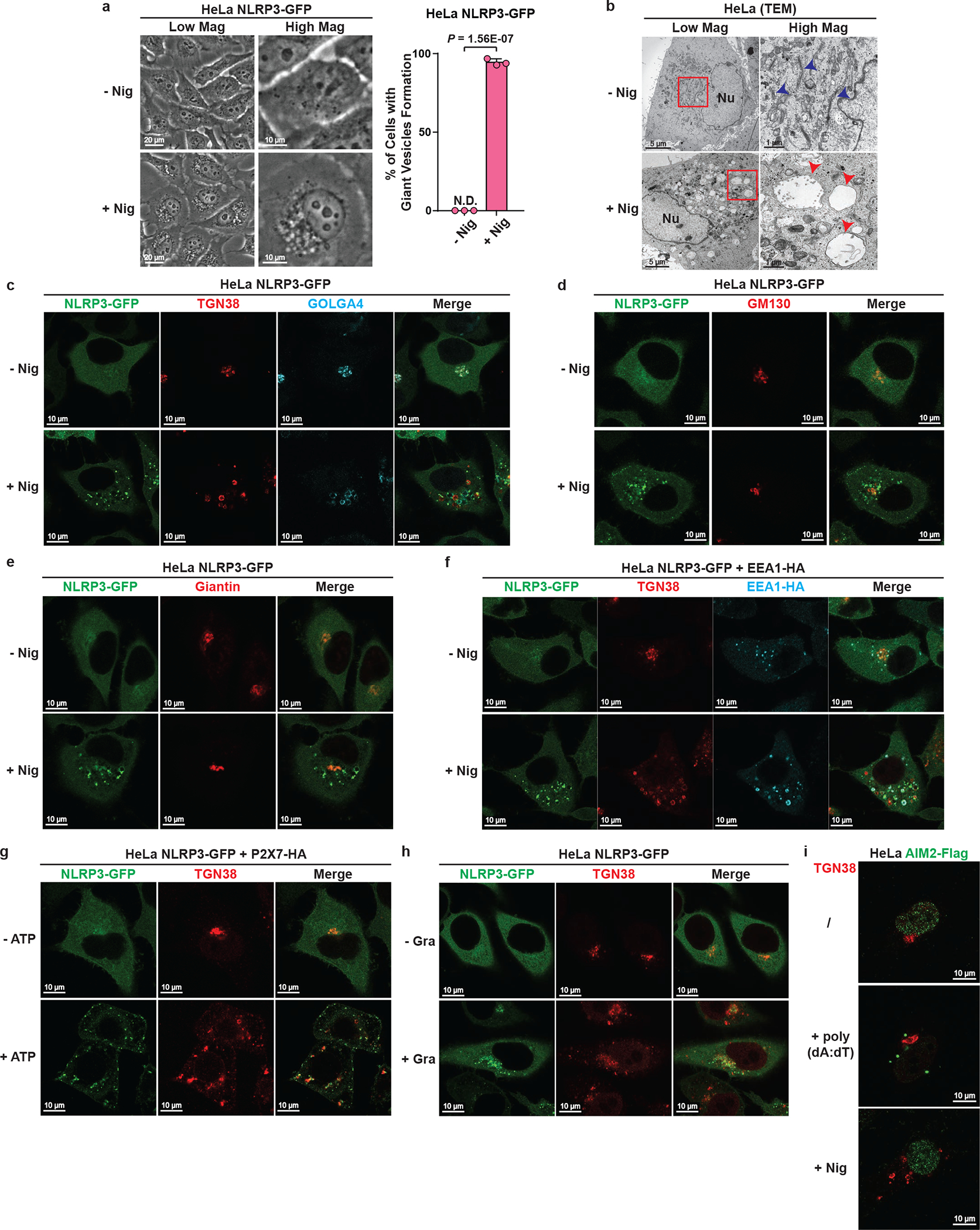
NLRP3 aggregates on stimulus-triggered dTGN. **a**, Nigericin treatment induced giant vesicles to form in the perinuclear region. HeLa NLRP3-GFP cells treated with or without nigericin (10 μM) for 80 min were examined under phase contrast microscope. Mag: magnification. Percentage of cells with giant vesicle formation was quantified from 100 cells (n = 3, mean ± SD, two-sided t test). N.D., not detectable. **b**, Ultrastructural analysis of nigericin-induced dispersed TGN (dTGN) vesicles. HeLa cells treated as in (**a**) were examined by transmission electron microscopy. Nu, nucleus. Blue arrowheads indicate Golgi stacks under resting conditions, while red arrowheads indicate nigericin-induced dTGN vesicles. Representative images from two independent experiments (more than 30 cells were examined for each condition in each experiment) are shown. **c**, Nigericin triggered the formation of dTGN, on which NLRP3 aggregated. HeLa cells stably expressing the indicated protein were stimulated as in (**a**) before immunostained for TGN38 and GOLGA4, two TGN markers. **d**, **e**, Cis- and medial-Golgi remained intact after nigericin treatment. Cells treated as in (**a**) were immunostained for GM130 (cis-Golgi maker) or giantin (cis-/medial-Golgi marker). **f**, NLRP3 aggregates mostly on dispersed TGN38-positive EEA1 but also on some EEA1-positive vesicles. HeLa cells stably expressing NLRP3-GFP and EEA1-HA were treated as in (**a**) before immunostained for TGN38 and HA (EEA1-HA). **g**, ATP stimulation led to NLRP3 aggregation on dTGN. HeLa cells stably expressing NLRP3-GFP and P2X7-HA were treated −/+ ATP (5 mM) for 80 min before imaging. P2X7 is a purinergic receptor essential for ATP-mediated NLRP3 inflammasome activation. **h**, Gramicidin stimulation led to NLRP3 aggregation on dTGN. HeLa cells were treated −/+ gramicidin (5 μM) for 80 min before imaging. **i**, DNA stimulation does not cause TGN dispersion or AIM2 recruitment to TGN. HeLa cells stably expressing AIM2-Flag were mock-transfected, transfected with poly(dA:dT) (1.5 μg/mL) for 3 hours or incubated with nigericin (10 μM) for 80 min before immunostained with antibodies against Flag (AIM2-Flag) or TGN38.

**Figure 2.**
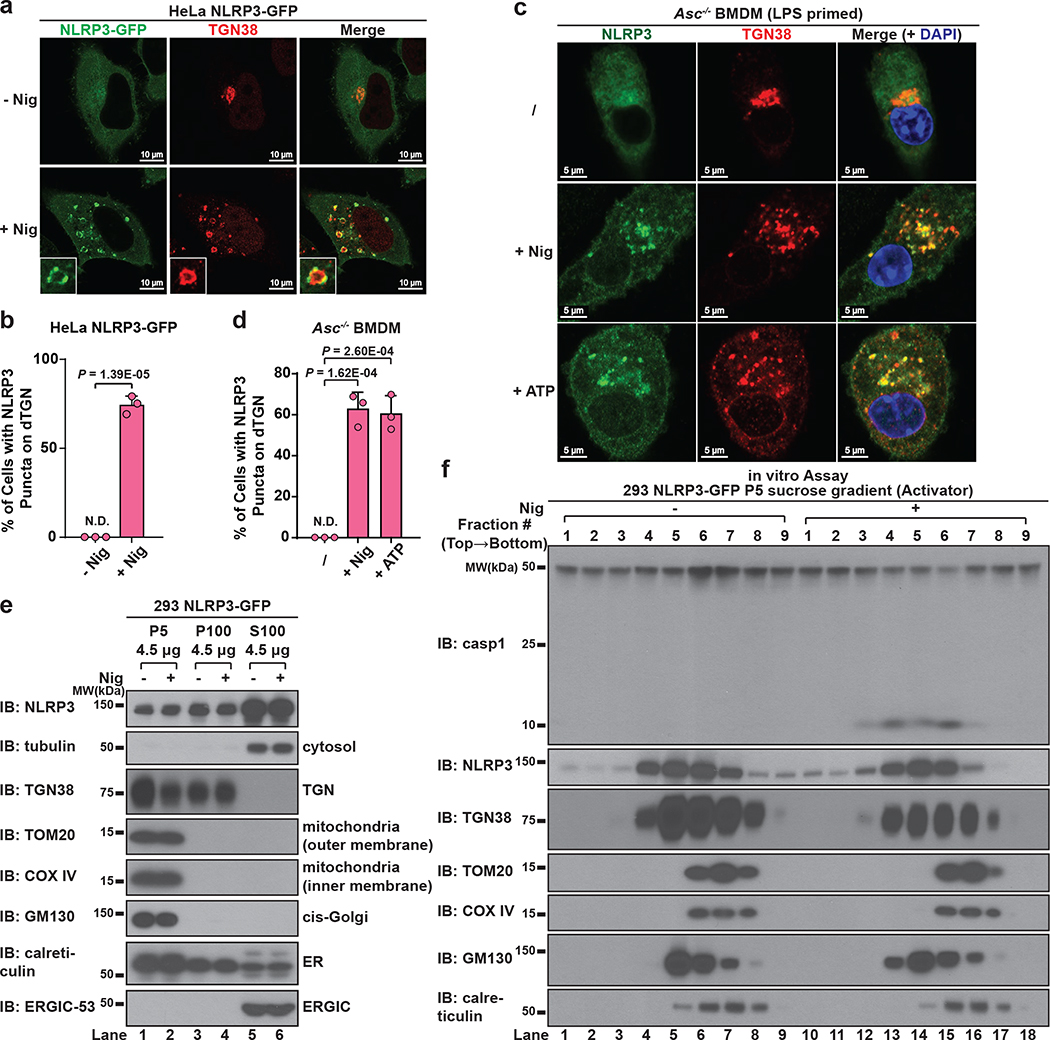
NLRP3 aggregates on dispersed TGN. **a**, **b**, Nigericin induced NLRP3 aggregation on dispersed TGN (dTGN) in HeLa cells stably expressing NLRP3-GFP. Percentage of cells with NLRP3 puncta on dTGN was quantified from 100 cells (n = 3, mean ± SD, two-sided t test). N.D., not detectable. **c**, **d**, Nigericin (10 μM) and ATP (5 mM) both induced endogenous NLRP3 aggregation on dTGN in primary ASC-deficient BMDMs. The cells were primed with LPS (50 ng/mL) for 3 hours. The data was collected and analyzed as in (**b**). **e**, Immunoblotting of indicated organelle markers in P5 (heavy membrane), P100 (light membrane) and S100 (cytosol) fractions. **f**, The P5 as shown in **e** was further fractionated by sucrose gradient ultracentrifugation followed by NLRP3 activity assay (top panel) or immunoblotting of each fraction (remaining panels).

We noticed that NLRP3 puncta were formed on the outside of vesicle-like structures (Fig. 1d and Supplementary Video 1). Indeed, in cells stimulated with nigericin, a number of giant vesicles appeared in the perinuclear region, typically 0.5–2 μm in diameter and composed of single membrane (Extended Data Fig. 2a–b). To understand where these vesicles are derived from, we examined various subcellular organelles in HeLa cells stably expressing NLRP3-GFP. Unexpectedly, the trans-face of Golgi complex (TGN) disassembled from a single perinuclear cluster into vesicles on which NLRP3 formed puncta (Fig. 2a–b and Supplementary Video 2).

Whereas the whole TGN disassembled into vesicles (Extended Data Fig. 2c), cis- and medial-Golgi still remained intact and didn’t colocalize with NLRP3 puncta (Extended Data Fig. 2d–e). There was no detectable change in other organelles examined (see Supplementary Information), with the only exception being the early endosomes (Extended Data Fig. 2f), probably due to their constant cargo transfer with TGN. Finally, both ATP and gramicidin, which are structurally unrelated to nigericin, also promoted TGN dispersion and NLRP3 recruitment (Extended Data Fig. 2g–h), although the sizes of dispersed TGN differed. In contrast, AIM2 activation didn’t involve either TGN disassembly or AIM2 translocation to TGN (Extended Data Fig. 2i). We hereinafter referred to the disassembled TGN triggered by NLRP3 stimuli collectively as dispersed TGN (dTGN).

**Extended Data Figure 3.**
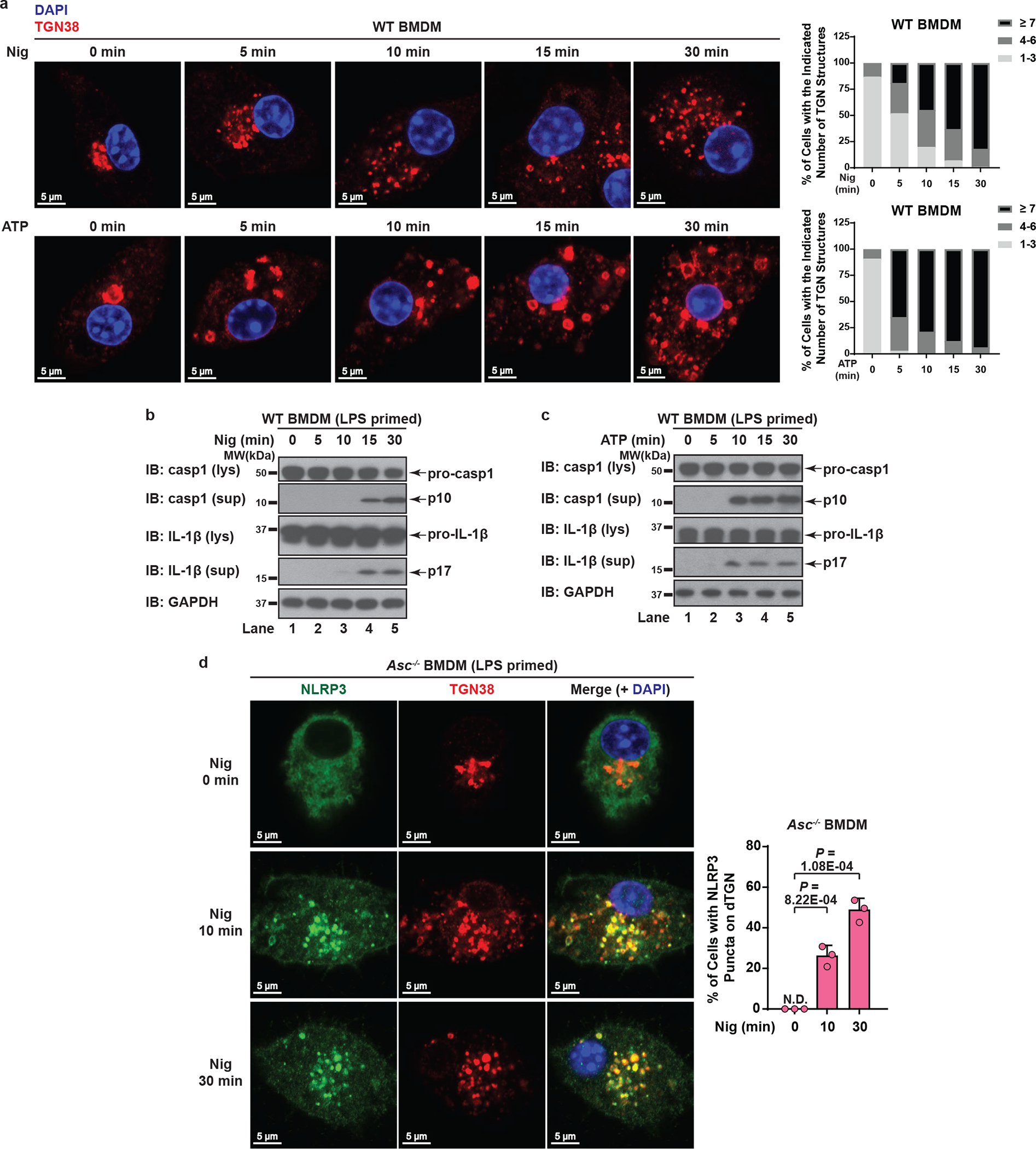
Endogenous NLRP3 is recruited to dTGN in primary macrophages. **a**, Dramatic TGN disassembly occurred at early time points in WT BMDMs. Cells were primed with LPS (50 ng/mL) for 3 hours, followed by nigericin (10 μM) or ATP (5 mM) stimulation for the indicated time and immunostained for TGN38. To quantify the level of TGN disassembly, the numbers of TGN structures not connected with each other for each cell were quantified from 100 randomly selected cells and grouped as shown in the right panels. **b**, **c**, Nigericin-induced caspase-1 and IL-1β cleavage didn’t occur until 15 min (for nigericin) or 10 min (for ATP) post stimulation in WT BMDMs. Cells were treated as in (**a**) before lysates were collected for immunoblotting. **d**, Endogenous NLRP3 aggregation on dTGN could be detected as early as 10 min post nigericin treatment in ASC-deficient BMDMs. Cells were primed with LPS (50 ng/mL) for 3 hours, followed by nigericin (10 μM) treatment for 0, 10 or 30 minutes before imaging. Percentage of cells with NLRP3 puncta on dTGN was quantified from 100 cells (n = 3, mean ± SD, two-sided t test). N.D., not detectable.

When primary wild type (WT) bone marrow-derived macrophages (BMDMs) were stimulated with either nigericin or ATP, dramatic TGN disassembly occurred and preceded caspase-1/IL-1β activation (Extended Data Fig. 3a–c). Importantly, when ASC-deficient primary BMDMs (to prevent the interference of ASC speck) were treated with nigericin or ATP, endogenous NLRP3 was recruited to dTGN and formed puncta (Fig. 2c–d). Notably, NLRP3 recruitment to dTGN in ASC-deficient BMDMs occurred before the earliest detectable caspase-1 and IL-1β cleavage in WT BMDMs with the same stimulation (Extended Data Fig. 3b and 3d, see Supplementary Information). Collectively, these results confirm that signal-dependent dTGN formation and endogenous NLRP3 recruitment also occur in physiologically relevant cells and precede the activation of NLRP3 inflammasome.

## NLRP3 activity is strongly associated with dTGN

**Extended Data Figure 4.**
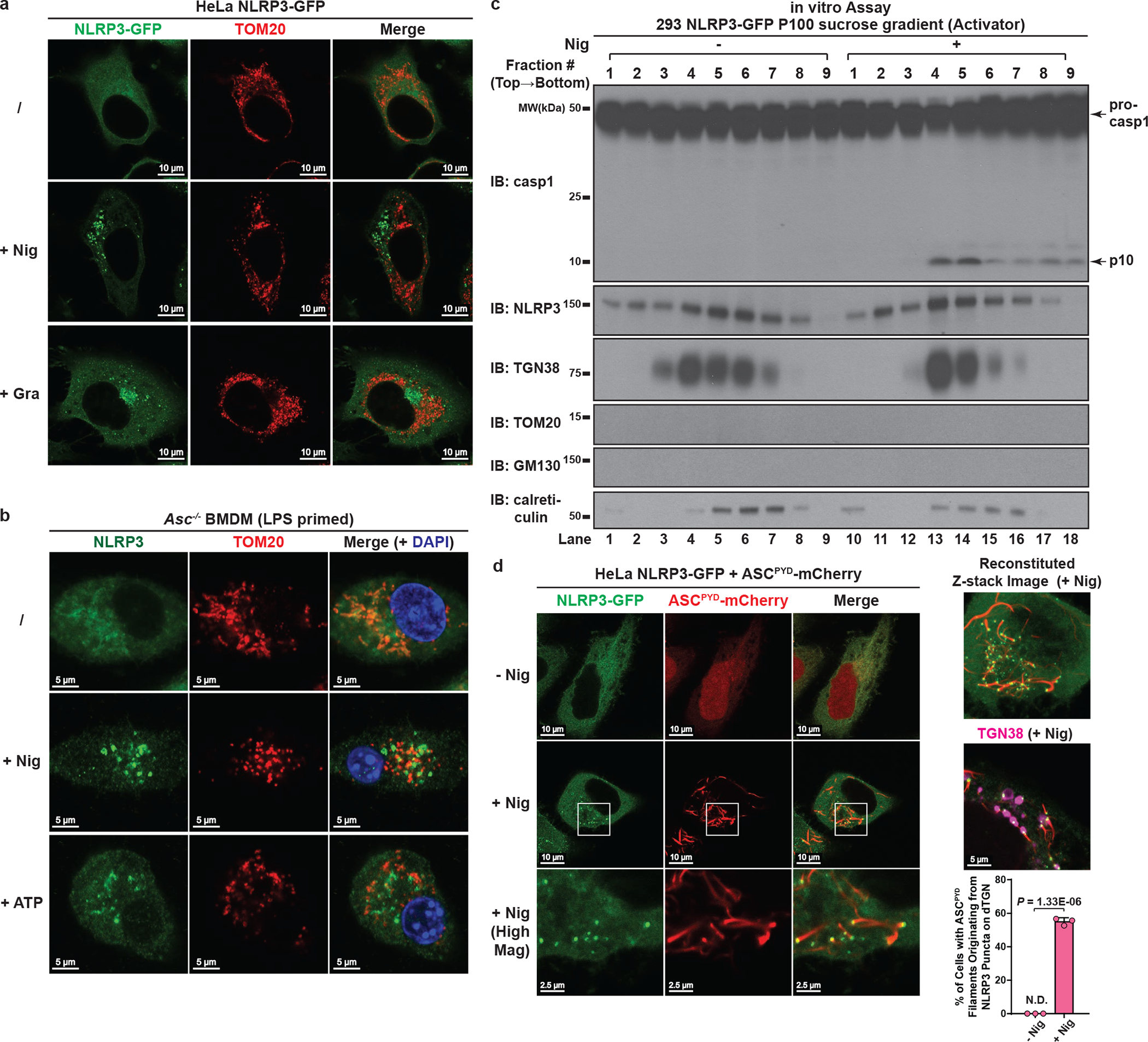
NLRP3 activity is strongly associated with dTGN but not mitochondria. **a**, NLRP3 did not translocate to mitochondria upon stimulation in HeLa cells. HeLa NLRP3-GFP cells were stimulated with nigericin (10 μM) or gramicidin (5 μM) for 80 min before immunostained for TOM20 (mitochondrial marker). **b**, Neither nigericin- nor ATP-induced NLRP3 puncta were colocalized with mitochondria in ASC-deficient BMDMs. Cells were primed with LPS (50 ng/mL) for 3 hours, followed by nigericin (10 μM) or ATP (5 mM) treatment for 60 min before immunostained for endogenous NLRP3 and TOM20. **c**, NLRP3 activity in P100 (light membrane) fraction was strongly associated with dTGN but not mitochondria. P100 fraction collected from Fig. 1c was subjected to sucrose gradient ultracentrifugation, before fractions were collected and examined by the in vitro NLRP3 activity assay (top panel). TOM20 (mitochondrial marker) and GM130 (cis-Golgi marker) were not detectable on immunoblots even after prolonged exposure. **d**, dTGN-localized NLRP3 puncta can initiate aggregation of ASC^PYD^. HeLa cells stably expressing the indicated proteins were incubated −/+ nigericin (10 μM) for 80 min before imaging. Mag, magnification. ASC^PYD^: aa 1–90 of murine ASC. Right panel (from top to bottom): reconstituted Z-stack image of a representative nigericin-treated cell; nigericin-treated cell co-immunostained for TGN38 (pseudo-colored in magenta); percentage of cells with ASC^PYD^ filaments originating from dTGN-localized NLRP3 puncta was quantified from 100 cells (n = 3, mean ± SD, two-sided t test). N.D., not detectable.

Previous literature has proposed that NLRP3 is activated via translocation to mitochondria, although this model has been challenged in recent studies^10,11^. We thus employed both imaging and biochemical analyses to further explore where active NLRP3 resides. Mitochondria had no detectable morphological change and didn’t colocalize with NLRP3 puncta after stimulation in either reconstituted HeLa cells (Extended Data Fig. 4a and Supplementary Video 3) or primary macrophages (Extended Data Fig. 4b), consistent with these puncta being localized on dTGN (Fig. 2a and 2c). In an independent approach, we used subcellular fractionation followed by the in vitro assay to examine the correlation between NLRP3 activity and organelle markers. Fig. 1c has shown that the majority of NLRP3 activity was present in P100 fraction, which was completely devoid of mitochondria (Fig. 2e). Indeed, mitochondria were only present in P5 fraction (Fig. 2e) and did not co-migrate with NLRP3 activity when P5 was further fractionated by sucrose gradient ultracentrifugation (Fig. 2f). In contrast, TGN was present in both P5 and P100 (Fig. 2e), and was the only organelle that strongly co-migrated with NLRP3 activity in both fractions (Fig. 2f and Extended Data Fig. 4c). Finally, we found that oligomerization of ASC^PYD^ was initiated from dTGN-localized NLRP3 puncta (Extended Data Fig. 4d, see Supplementary Information). Together, these data strongly indicate that NLRP3 is recruited to dTGN to become active.

## NLRP3 is recruited to dTGN via its polybasic region

**Extended Data Figure 5.**
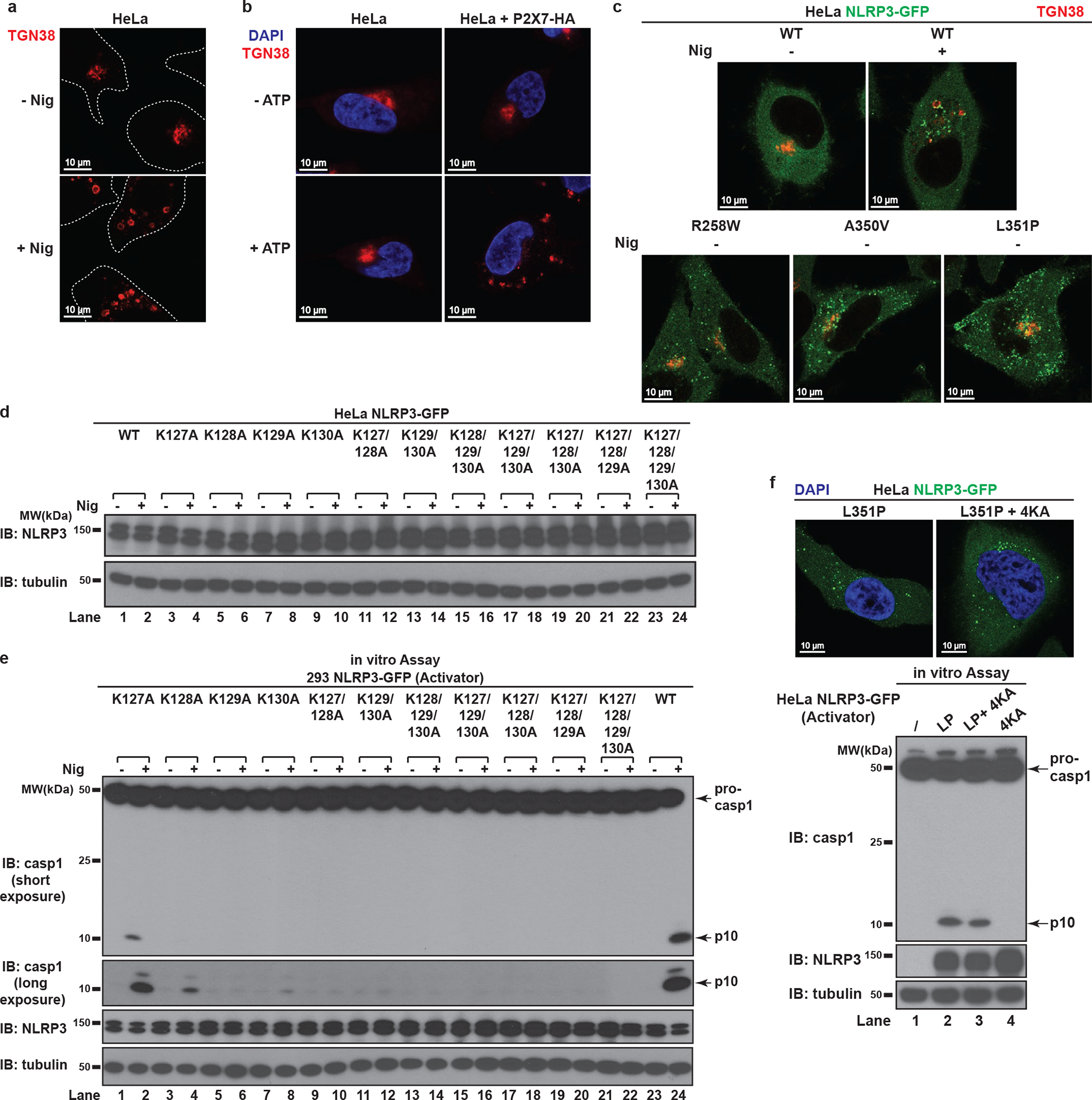
The polybasic region mediates the dTGN recruitment and activation of NLRP3. **a**, NLRP3 is not required for dTGN formation in response to nigericin in HeLa. HeLa cells (without NLRP3 expression) were treated −/+ nigericin (10 μM) for 80 min and immunostained for TGN38. Cell borders are outlined with dashed lines. **b**, ATP induced dTGN formation in a manner dependent on P2X7 but not NLRP3. HeLa cells with or without P2X7-HA stable expression were incubated −/+ ATP (5 mM) for 80 min before immunostained for TGN38. **c**, Constitutively active mutants of NLRP3 can bypass the step of TGN recruitment by spontaneously forming aggregates in the cytosol. Cells stably expressing the indicated proteins were treated −/+ nigericin (10 μM) for 80 min before immunostained for TGN38. **d**, Immunoblots for cells used in Fig. 3c. **e**, The KKKK motif is critical for nigericin-induced NLRP3 activation. Cells stably expressing the indicated proteins were treated −/+ nigericin (10 μM) for 60 min before examined with the in vitro NLRP3 activation assay. **f**, Mutations in the KKKK motif do not compromise the ability of constitutively active NLRP3 to activate caspase-1. Cells stably expressing the indicated proteins were examined by fluorescence microscopy (upper panel) and the in vitro NLRP3 activity assay (lower panel) without any stimulation. LP, L351P; 4KA, K127/128/129/130A.

dTGN formation occurred earlier than NLRP3 puncta formation (Supplementary Video 2), and is actually independent of NLRP3 (Extended Data Fig. 5a–b and Supplementary Video 4), suggesting that the presence of dTGN may be a prerequisite for the recruitment, aggregation and activation of NLRP3. This is consistent with the observation that the constitutively active NLRP3 disease mutants could bypass TGN recruitment since they were able to aggregate without stimulation (Extended Data Fig. 5c).

**Figure 3.**
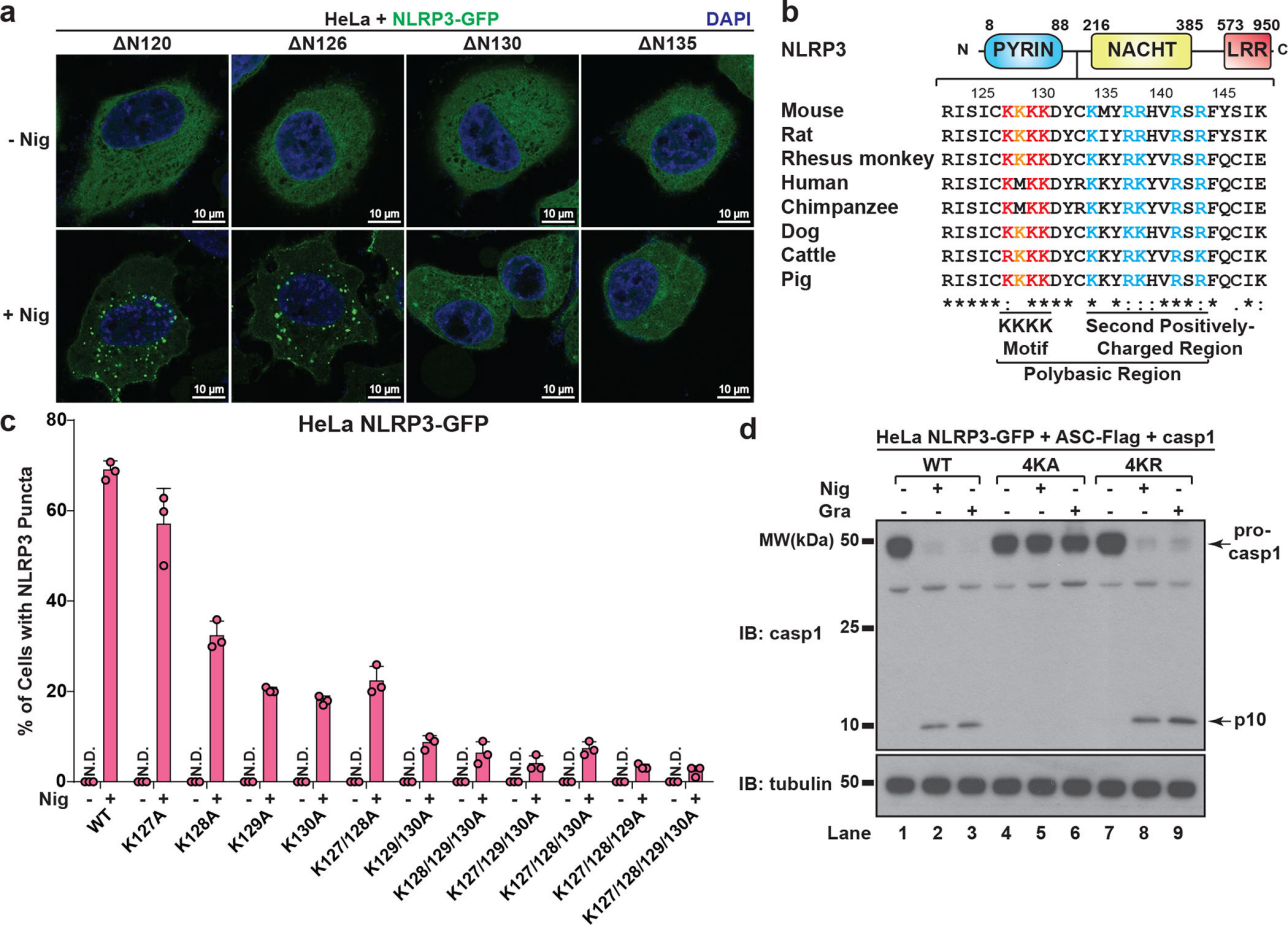
NLRP3 is recruited to dTGN via its polybasic region. **a**, HeLa cells stably expressing the N-terminally truncated NLRP3 proteins were stimulated −/+ nigericin. **b**, The polybasic region of NLRP3 is highly conserved in all identified NLRP3 orthologs (aligned using Clustal Omega). **c**, Cells stably expressing the indicated proteins were treated as in (**a**), before percentage of cells with NLRP3 puncta was quantified from 100 cells (n = 3, mean ± SD). N.D., not detectable. **d**, HeLa cells stably expressing NLRP3 WT, 4KA or 4KR and other indicated proteins were treated −/+ nigericin or gramicidin before immunoblotting. 4KA, K127/128/129/130A; 4KR, K127/128/129/130R.

To investigate how NLRP3 is recruited to dTGN, we examined several N-terminally truncated mutants of NLRP3 and found that deletion of four consecutive lysine residues (amino acid 127 to 130, hereinafter referred to as the KKKK motif) abruptly abolished NLRP3 aggregation (Fig. 3a). The KKKK motif is a highly conserved region between pyrin domain and NACHT domain, with at least three positively-charged residues present in all currently known NLRP3 orthologs (Fig. 3b). Strikingly, mutations of the KKKK motif to alanine inhibited the ability of full-length NLRP3 to form puncta in a manner dependent on the number of remaining lysine residues, with K127/128/129/130A (“4KA”) mutant having almost no detectable puncta (Fig. 3c and Extended Data Fig. 5d). All NLRP3 mutants that were compromised in forming puncta also had defective activity (Extended Data Fig. 5e). As a control, 4KA mutations didn’t affect the ability of the constitutively active mutant L351P to aggregate in the cytosol or activate downstream signaling without stimulation (Extended Data Fig. 5f), indicating that 4KA didn’t block the interaction of NLRP3 with ASC directly.

**Extended Data Figure 6.**
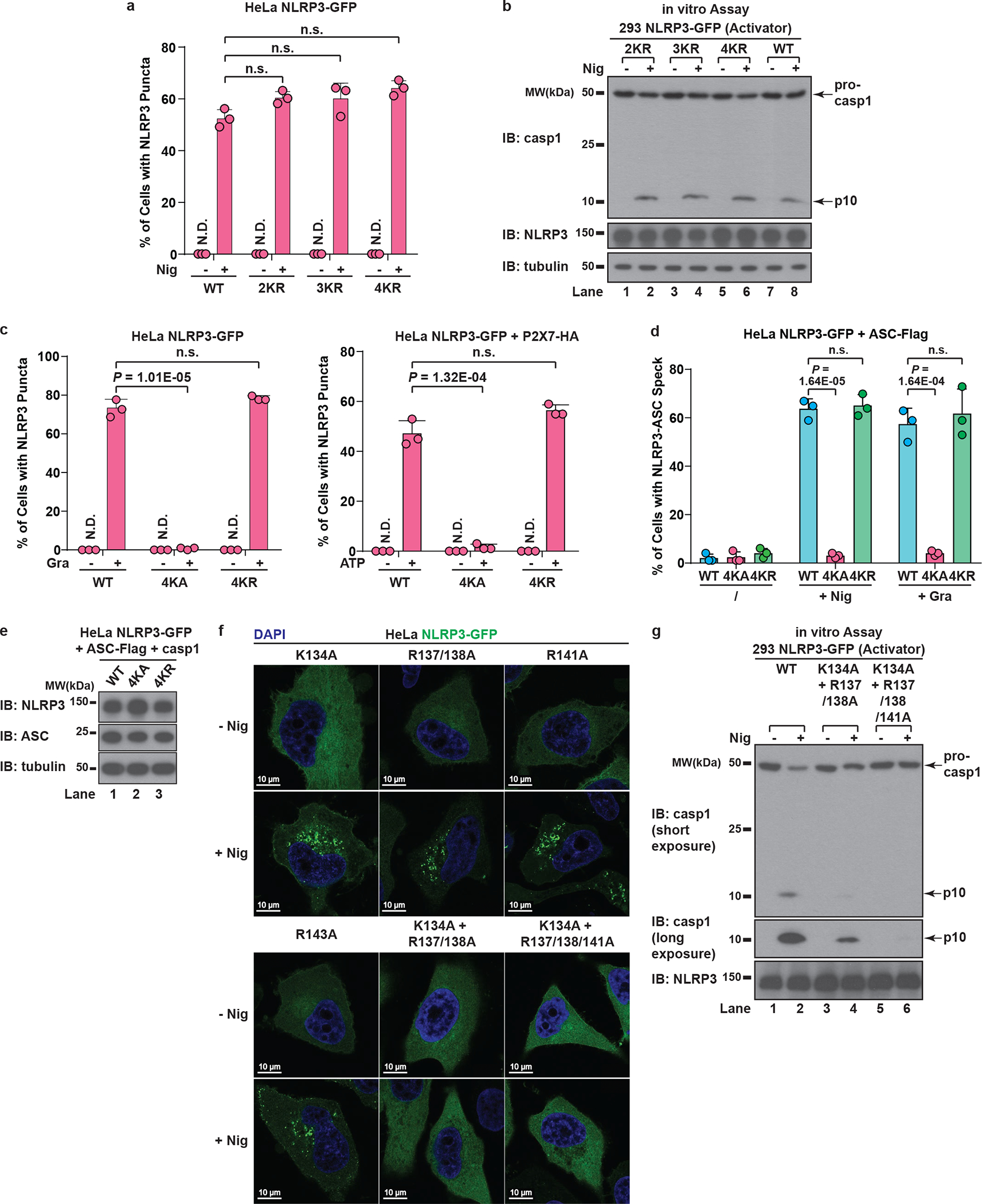
The polybasic region of NLRP3 functions through its positive charge. **a**, Mutations of the KKKK motif to arginine do not affect nigericin-induced NLRP3 puncta formation. HeLa cells stably expressing the indicated proteins were treated −/+ nigericin (10 μM) for 80 min before imaging. The percentage of cells with NLRP3 puncta was quantified from 100 cells (n = 3, mean ± SD, two-sided t test). N.D., not detectable. n.s., not significant (alpha = 0.01). 2KR, K127/128R; 3KR, K127/128/129R; 4KR, K127/128/129/130R. **b**, Mutations of the KKKK motif to arginine do not impair nigericin-induced NLRP3 activation. Cells stably expressing the indicated proteins were treated −/+ nigericin (10 μM) for 60 min before cell extracts were examined by the in vitro NLRP3 activity assay. **c**, The positive charge of the KKKK motif is important for gramicidin- and ATP-induced NLRP3 puncta formation. HeLa cells stably expressing the indicated proteins were treated −/+ gramicidin (5 μM) (left panel) or ATP (5 mM) (right panel) for 80 min, before the percentage of cells with NLRP3 puncta was analyzed as in (**a**). **d**, The positive charge of the KKKK motif is essential for NLRP3 to polymerize ASC. Cells stably expressing the indicated proteins were treated −/+ nigericin (10 μM) or gramicidin (5 μM) for 80 min before immunostained for both NLRP3 and ASC. The percentage of cells with NLRP3-ASC speck was analyzed as in (**a**). **e**, Immunoblots for cells used in Fig. 3d. **f**, **g**, The second positively-charged region of NLRP3 is also important for its aggregation on dTGN and activation in a manner dependent on the number of remaining positively-charged residues. Cells stably expressing the indicated proteins were treated −/+ nigericin (10 μM) before imaging (**f**) or examined by the in vitro NLRP3 activity assay (**g**).

When the KKKK motif was mutated to arginine (“4KR”), neither the puncta formation nor activation of NLRP3 by nigericin was affected (Extended Data Fig. 6a–b), indicating that the positive charge of the motif is critical for NLRP3 recruitment and activation. Similarly, 4KA but not 4KR was largely defective in puncta formation in response to gramicidin and ATP (Extended Data Fig. 6c). Finally, 4KA but not 4KR lost the ability to induce ASC oligomerization (Extended Data Fig. 6d) and caspase-1 cleavage in the cells (Fig. 3d and Extended Data Fig. 6e). We also identified a second positively-charged region located after the KKKK motif (Fig. 3b), which is also important for the recruitment and activation of NLRP3 (Extended Data Fig. 6f–g, see Supplementary Information). Taken together, these results demonstrate that the conserved polybasic region including the KKKK motif serves a critical function in recruiting NLRP3 to dTGN upon stimulation, which is essential for its subsequent activation.

## NLRP3 is recruited to dTGN via binding to PI4P

**Extended Data Figure 7.**
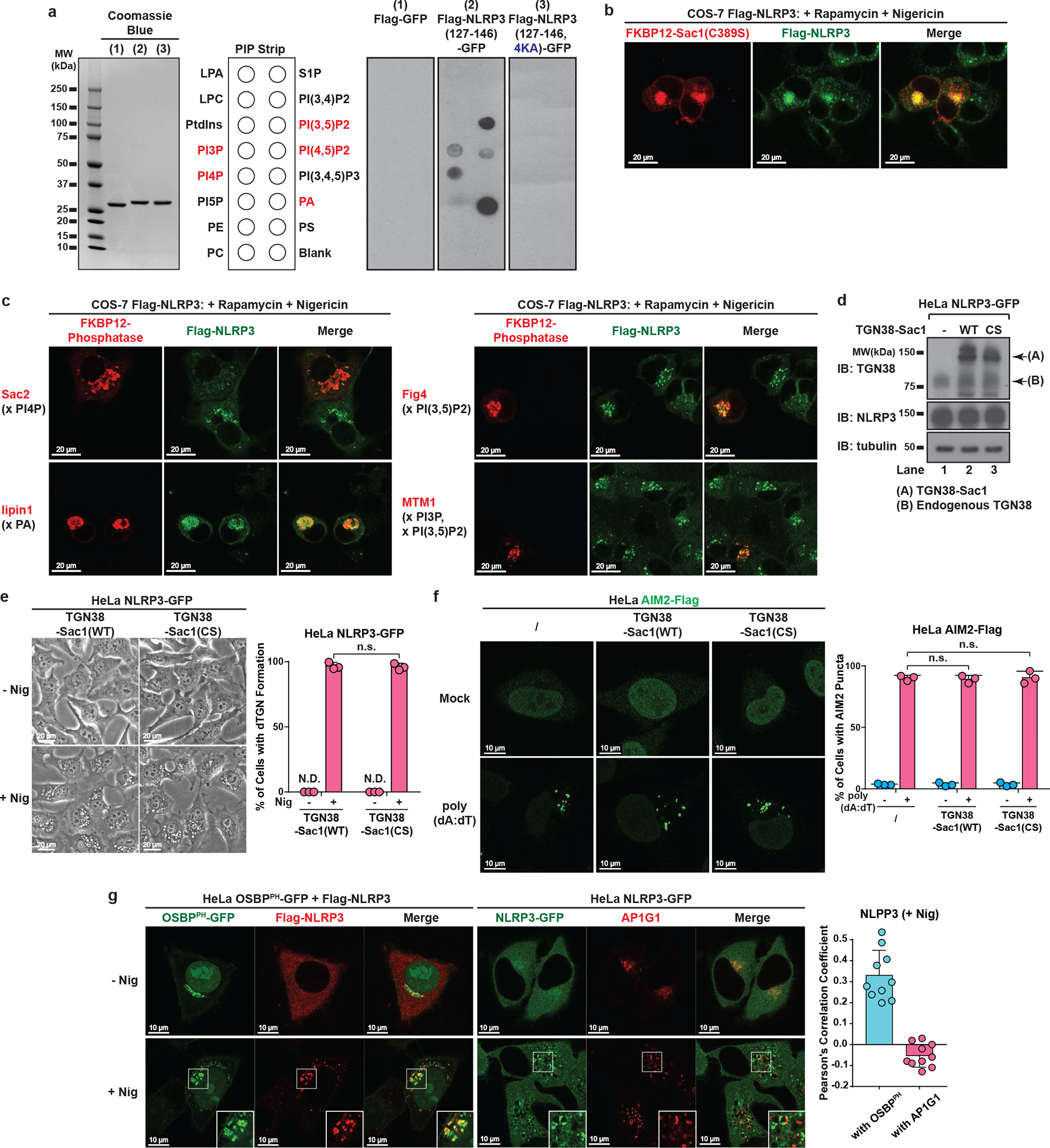
NLRP3 is recruited to dTGN via binding to PI4P. **a**, The polybasic region of NLRP3 interacted with phospholipids in vitro through its positive charge. Left panel: Coomassie Blue staining of purified proteins of interest, with Flag-GFP as a control. Right panel: PIP Strip membranes blotted with various lipids were incubated sequentially with proteins of interest, Flag antibody and HRP secondary antibody before exposure. Phospholipids with positive binding on the second PIP Strip are highlighted in red. **b**, Catalytically inactive Sac1 did not impair the recruitment of NLRP3 to dTGN. COS-7 cells stably expressing Flag-NLRP3 were transiently transfected with TGN38-FRB and mRFP-FKBP12-Sac1(C389S), incubated with rapamycin (1 μM) and nigericin (10 μM) for 80 min before imaging. Note that nigericin-induced dTGN in COS-7 cells sometimes look like a single cluster because of the relatively small size of COS-7 cells and the high intensity of fluorescence signal. Under phase contrast microscope these dTGN were separated vesicles adjacent to each other (data not shown). **c**, Only PI4P phosphatase blocked NLRP3 recruitment to dTGN. Similar to (**b**), except that indicated phosphatases were used. The target phospholipids are labeled after “x”. **d**, Immunoblots of HeLa NLRP3-GFP cells stably expressing TGN38-Sac1 (WT or C389S). CS, C389S. **e**, TGN-targeted Sac1 didn’t affect general cell morphology or nigericin-induced dTGN formation. Cells were treated −/+ nigericin (10 μM) for 80 min before imaged under phase contrast microscope. The percentage of cells with dTGN formation was quantified from 100 cells (n = 3, mean ± SD, two-sided t test). N.D., not detectable. n.s., not significant (alpha = 0.01). **f**, TGN-targeted Sac1 didn’t affect AIM2 aggregation. Cells stably expressing the indicated proteins were transfected with or without poly(dA:dT) (1.5 μg/mL) for 3 hours before imaging. The percentage of cells with AIM2 aggregates was analyzed as in (**e**). **g**, NLRP3 puncta were restricted to PI4P-enriched microdomains. Cells stably expressing the indicated proteins were treated −/+ nigericin (10 μM) for 80 min before imaging. Inset: higher magnification of dTGN. Colocalization analysis was performed by calculating Pearson’s correlation coefficient (threshold regression: Costes) using Coloc 2 plugin of ImageJ. Data are presented as mean ± SD.

**Figure 4.**
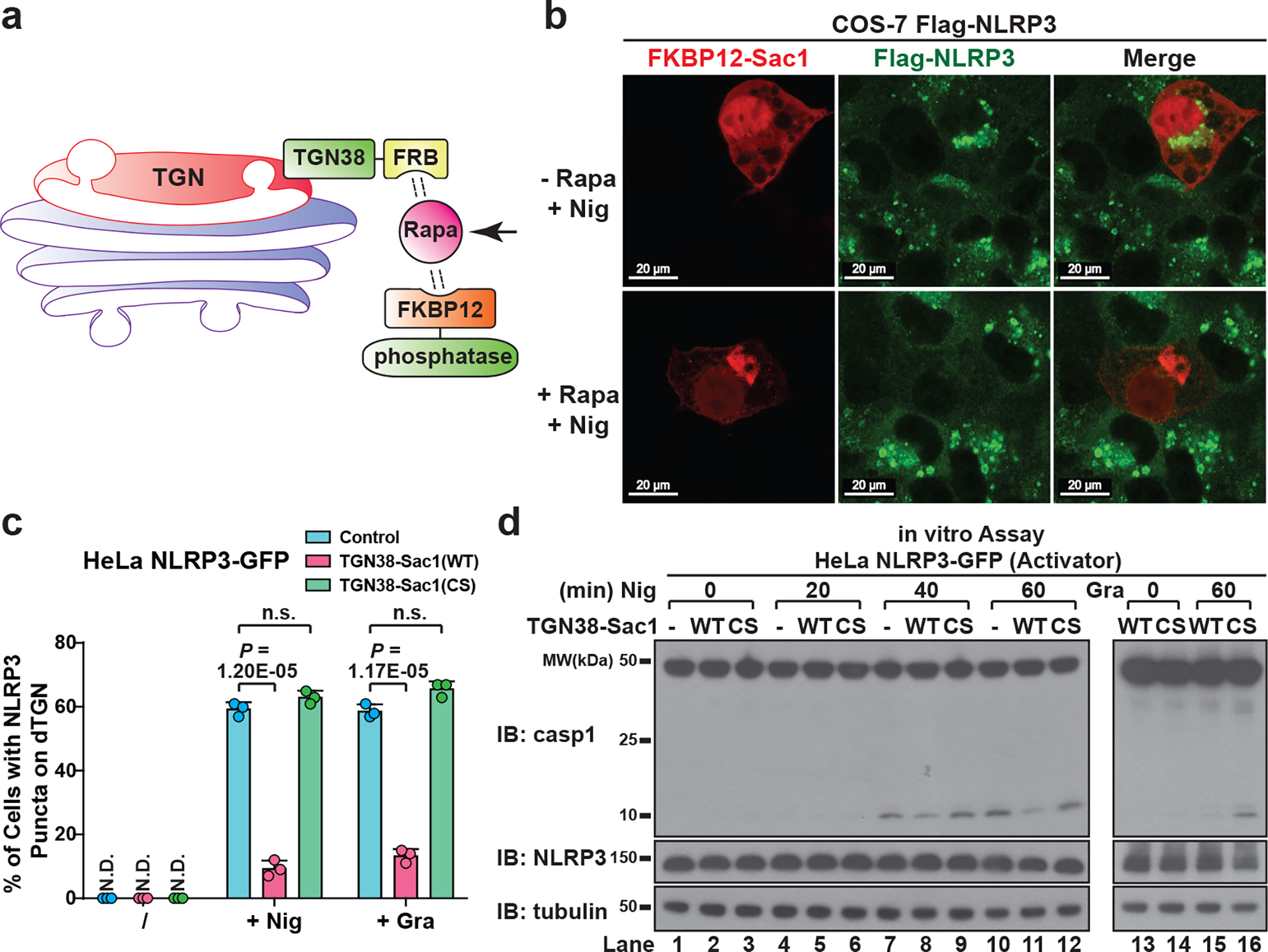
NLRP3 is recruited to dTGN via binding to PI4P. **a**, Inducible recruitment system of phosphatases: the addition of rapamycin (Rapa) (1 μM) induced the heterodimerization of FRB and FKBP12, thus recruiting the phosphatase to TGN to hydrolyze its target phospholipid. **b**, Inducible translocation of PI4P phosphatase Sac1 to TGN inhibited nigericin-induced NLRP3 puncta formation. Arrows indicate cells with Sac1 expression. **c**, HeLa cells stably expressing the indicated proteins were treated −/+ nigericin or gramicidin. The percentage of cells with NLRP3 puncta was quantified from 100 cells (n = 3, mean ± SD, two-sided t test). N.D., not detectable. n.s., not significant (alpha = 0.01). CS, C389S. **d**, Lysates from the cells treated as in (**c**) were analyzed by the in vitro NLRP3 activity assay.

Several Rab GTPases are known to use polybasic regions to bind to negatively-charged phospholipids PI(3,4,5)P3 and PI(4,5)P2 on plasma membrane^12^. To test whether the polybasic region in NLRP3 mediates its recruitment through a similar mechanism, we purified an NLRP3 fragment containing the polybasic region (aa 127–146) and tested its binding to phospholipids via lipid blot assay. Interestingly, this fragment bound to several negatively-charged phospholipids, which was abolished with 4KA mutations (Extended Data Fig. 7a). We next examined which phospholipid is important for recruitment of full-length NLRP3 to dTGN in live cells, by taking advantage of the recently developed inducible recruitment system of phospholipid phosphatases^13^, in which the addition of rapamycin promotes the heterodimerization of FKBP12 and FRB, thus recruiting the phosphatase to TGN where it hydrolyzes its target phospholipid (Fig. 4a). Sac1, a PI4P phosphatase, abolished NLRP3 recruitment to dTGN in a manner dependent on the translocation of Sac1 to TGN (Fig. 4b) and its catalytic activity (Extended Data Fig. 7b). Another PI4P phosphatase Sac2^14^ also abolished NLRP3 recruitment, whereas phosphatases targeting other phospholipids had no detectable effect on NLRP3 recruitment (Extended Data Fig. 7c).

To better quantify the impact of PI4P on NLRP3 recruitment and activation, we stably expressed TGN38-Sac1 fusion protein in HeLa NLRP3-GFP cells (Extended Data Fig. 7d). This didn’t affect the general cellular morphology or nigericin-induced dTGN formation (Extended Data Fig. 7e), but largely impaired NLRP3 puncta formation through its phosphatase activity (Fig. 4c). As a control, TGN38-Sac1 didn’t affect poly(dA:dT)-induced AIM2 aggregation (Extended Data Fig. 7f). Moreover, nigericin-induced NLRP3 puncta had strong colocalization with the PH domain of OSBP (OSBP^PH^), one of the best characterized PI4P-binding domains^15^, but not with the AP-1 complex (Extended Data Fig. 7g) that mainly relies on ARF1 for TGN targeting^16,17^, again showing that NLRP3 is specifically recruited to PI4P-enriched microdomains on TGN. Finally, TGN38-Sac1 significantly repressed NLRP3 activation through its catalytic activity (Fig. 4d). Together, these results show that PI4P binding is important for NLRP3 recruitment to dTGN and its subsequent activation, which is in agreement with the previous observation that PI4P is the dominant phosphoinositide on TGN^18^.

**Extended Data Figure 8.**
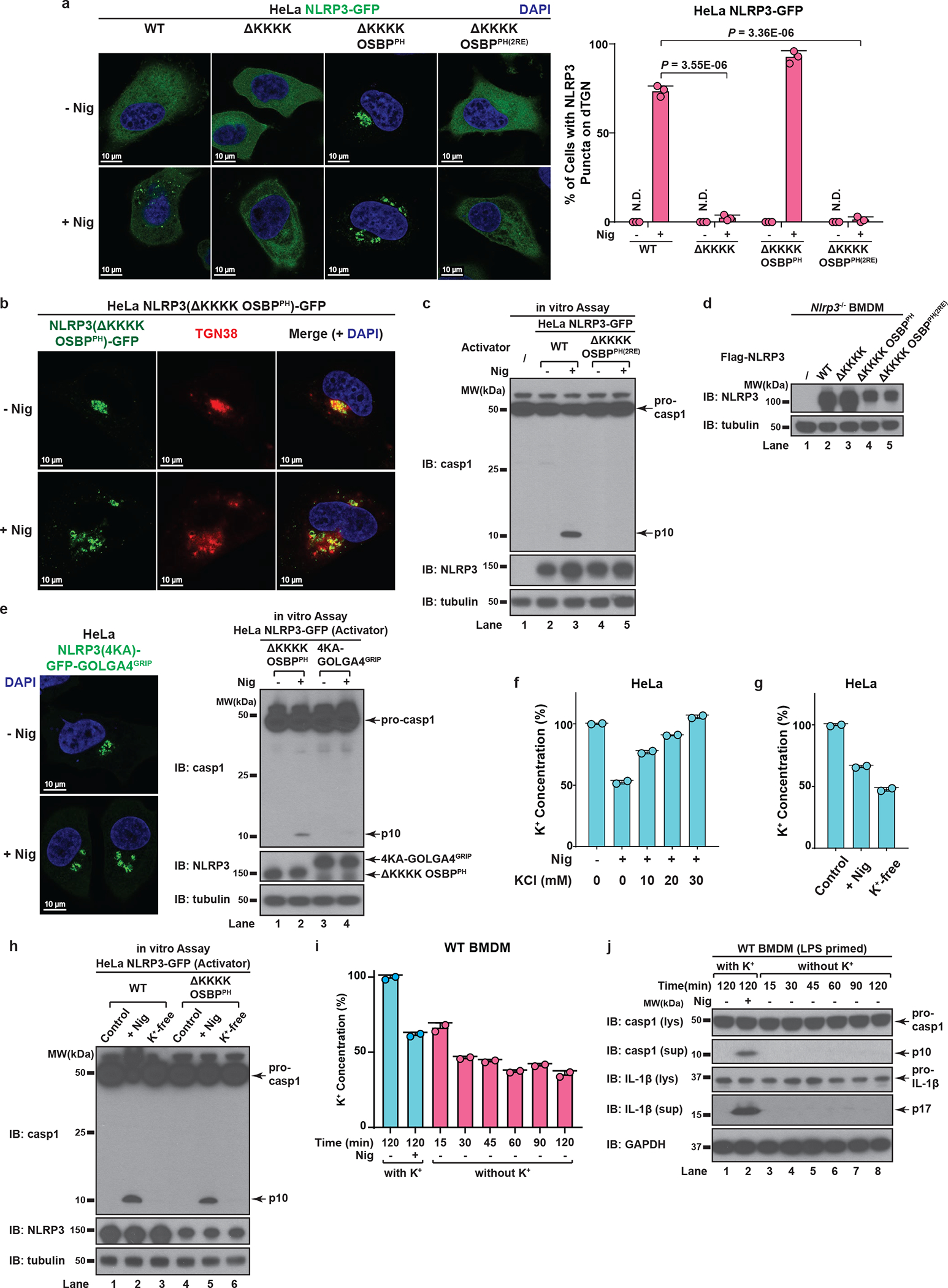
Binding to PI4P on dTGN is essential for NLRP3 inflammasome activation. **a**, **b**, The KKKK motif of NLRP3 can be functionally replaced with a PI4P-binding domain of OSBP. (**a**) Cells stably expressing the indicated proteins were treated −/+ nigericin (10 μM) for 80 min before imaging. The percentage of cells with NLRP3 on dTGN was quantified from 100 cells (n = 3, mean ± SD, two-sided t test). N.D., not detectable. 2RE, R107/108E. Note that NLRP3(ΔKKKK OSBP^PH^) constitutively localized on intact TGN without stimulation was not counted in the quantification. (**b**) Replacement of the KKKK motif with OSBP^PH^ domain allowed NLRP3 to be constitutively localized on TGN. Cells were treated as in (**a**) before immunostained for TGN38. **c**, Mutations of OSBP PH domain that abrogates its PI4P binding also abolish its ability to functionally rescue NLRP3(ΔKKKK). Cells were treated as in (**a**) before extracts were examined by the in vitro NLRP3 activity assay. **d**, Immunoblots for primary NLRP3-deficient BMDMs rescued with Flag-NLRP3 (WT or mutants), which were used for experiment in Fig. 5b and 6**d**. The cells were infected with lentivirus encoding the indicated proteins for 6 days before immunoblotting. **e**, Recruitment of NLRP3 to non-PI4P-enriched regions of TGN is not sufficient to support its activation. Cells stably expressing the indicated proteins were treated as in (**a**) before examined by fluorescence microscopy (left panel; images for NLRP3(ΔKKKK OSBP^PH^) are shown in (**a**)) and the in vitro assay (right panel). **f**, Extracellular KCl at 30 mM was sufficient to completely block nigericin-induced K^+^ efflux. Cells were treated −/+ nigericin (10 μM) for 80 min in the presence of increasing concentrations of KCl, before cell extracts were collected for measurement of intracellular K^+^ concentration (shown as percentage change compared to untreated sample, mean ± SD). Representative results from two independent experiments (each contains two samples for each condition) are shown. **g**, Incubation in K^+^-free medium induced spontaneous K^+^ efflux. Cells were incubated in Hanks buffer containing 5 mM K^+^ for the first two conditions, or Hanks buffer without K^+^ (replaced by Na^+^) for the third condition (K^+^-free). The second condition also contained nigericin (10 μM). After 80 min, the cell extracts were collected for intracellular K^+^ measurement with methods similar to (**f**). **h**, K^+^ efflux alone is not sufficient to activate either WT NLRP3 or NLRP3(ΔKKKK OSBP^PH^). Cells stably expressing the indicated proteins were treated as in (**g**) before examined by the in vitro NLRP3 activity assay. **i**, Incubation in K^+^-free medium induced spontaneous K^+^ efflux in primary WT BMDMs. Cells were primed with LPS (50 ng/mL) for 3 hours, before treatment as in (**g**) with the indicated time lengths. Intracellular K^+^ concentrations were then measured and analyzed as in (**f**). **j**, K^+^ efflux alone is not sufficient to activate endogenous NLRP3 in primary WT BMDMs. Cells treated as in (**i**) were used for immunoblotting. lys, lysate; sup, supernatant.

**Figure 5.**
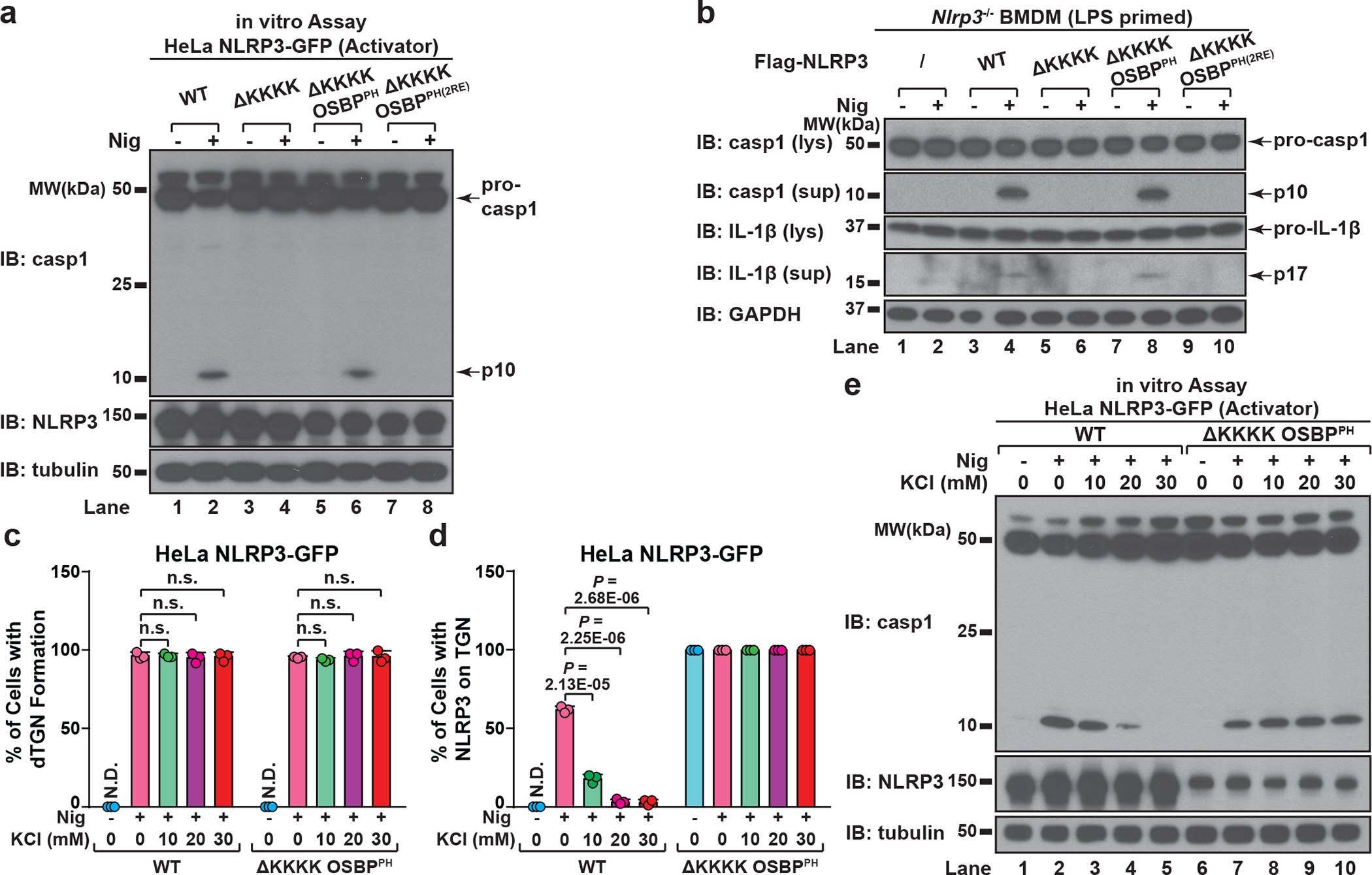
PI4P binding by NLRP3 is essential for the inflammasome activation **a**, HeLa cells stably expressing the indicated proteins were treated −/+ nigericin before examined by the in vitro NLRP3 activity assay. 2RE, R107/108E. **b**, Primary NLRP3-deficient BMDMs reconstituted with the indicated proteins were primed with LPS before nigericin stimulation. Cell lysates (lys) and supernatants (sup) were analyzed by immunoblotting to detect cleaved caspase-1 and IL-1b. **c**, Cells were treated −/+ nigericin in the presence of increasing concentrations of KCl, before the percentage of cells with dTGN formation was quantified from 100 cells (n = 3, mean ± SD, two-sided t test). N.D., not detectable. n.s., not significant (alpha = 0.01). **d**, Cells were treated as in (**c**), before the percentage of cells with NLRP3 on TGN (either intact or forming dTGN) was analyzed as in (**c**). **e**, Cells treated as in (**c**) and the cell lysates were analyzed by the in vitro NLRP3 activity assay.

As expected, deletion of the KKKK motif (ΔKKKK) abolished the recruitment of NLRP3 to dTGN, while replacement of the KKKK motif with OSBP^PH^ (ΔKKKK OSBP^PH^) constitutively targeted NLRP3 to TGN (Extended Data Fig. 8a–b). In contrast, insertion of OSBP^PH(R107/108E)^, which harbors mutations that abolish its binding to PI4P^19^, did not allow NLRP3(ΔKKKK) to translocate to TGN (Extended Data Fig. 8a). Importantly, NLRP3(ΔKKKK) showed little activity after stimulation, which could be rescued by the insertion of OSBP^PH^ but not OSBP^PH(R107/108E)^ in both reconstituted HeLa cells and primary NLRP3-deficient BMDMs (Fig. 5a–b and Extended Data Fig. 8c–d). Notably, targeting to PI4P-enriched microdomains rather than general TGN localization is essential for NLRP3 activation (Extended Data Fig. 8e, see Supplementary Information). These results thus confirm that the KKKK motif serves as a PI4P binding domain for NLRP3 recruitment and activation.

To test whether potassium (K^+^) efflux, a cellular signal previously shown to be important for NLPR3 activation^20^, is required for dTGN formation, NLRP3 recruitment and/or TGN-localized NLRP3 to become active, we examined the sensitivity of WT NLRP3 and NLRP3(ΔKKKK OSBP^PH^) to increasing concentrations of KCl in extracellular medium, which abolished stimulus-induced K^+^ efflux^20^ (Extended Data Fig. 8f). K^+^ efflux inhibition had no effect on dTGN formation (Fig. 5c), indicating that dTGN formation doesn’t require K^+^ efflux. Surprisingly, while K^+^ efflux inhibition significantly repressed the recruitment of WT NLRP3 to dTGN and blocked its activation, it had no effect on either the constitutive TGN localization or signal-dependent activation of NLRP3(ΔKKKK OSBP^PH^) (Fig. 5d–e). This indicates that K^+^ efflux is essential for WT NLRP3 recruitment to TGN, probably by lowering cellular ionic strength to promote the ionic binding; however, once NLRP3 is recruited to TGN, e.g. in the case of NLRP3(ΔKKKK OSBP^PH^), it no longer requires K^+^ efflux for subsequent activation. Interestingly, even though NLRP3(ΔKKKK OSBP^PH^) was targeted to TGN constitutively and no longer required K^+^ efflux, its activation was still signal-dependent. This likely indicates that binding to PI4P on dispersed TGN instead of intact TGN is essential for NLRP3 to become active. This is consistent with the observation that spontaneous K^+^ efflux alone is not sufficient for NLRP3 activation in both reconstituted HeLa cells (Extended Data Fig. 8g–h) and primary BMDMs (Extended Data Fig. 8i–j), since there was no stimulus-induced TGN dispersion.

## PI4P enables K^+^ efflux-independent NLRP3 activation

**Extended Data Figure 9.**
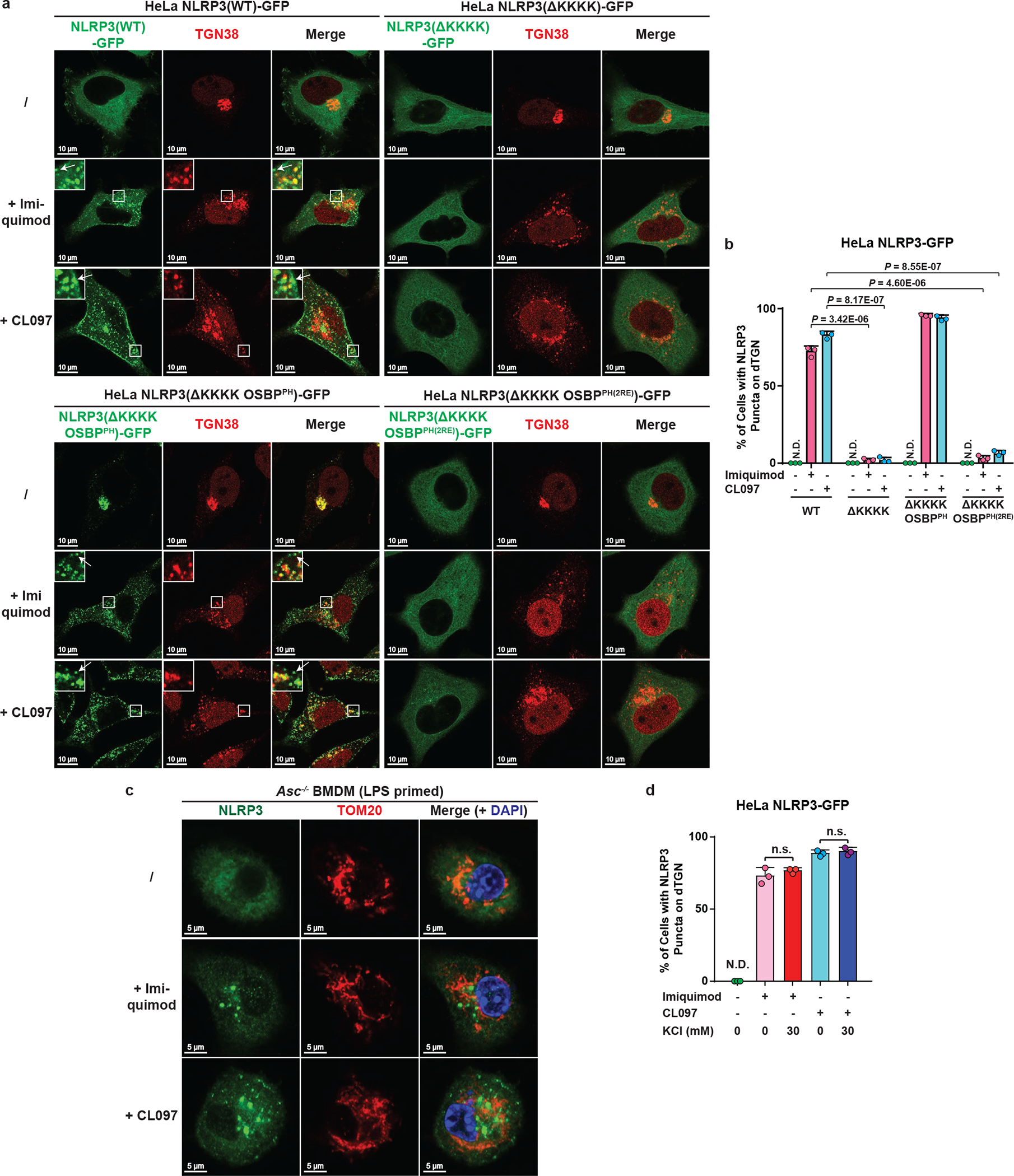
K^+^ efflux-independent stimuli also induced TGN dispersion and PI4P-dependent NLRP3 recruitment. **a**, **b**, K^+^ efflux-independent stimuli also induced NLRP3 aggregation on dTGN through the KKKK motif. HeLa cells stably expressing the indicated proteins were treated −/+ imiquimod or CL097 (45 μg/mL) for 80 min before imaging. High magnification images are shown in the inset. Arrows indicate representative plasma-membrane-localized NLRP3 puncta, which were separated from TGN38-positive compartments due to the partial separation of PI4P and TGN38. The percentage of cells with NLRP3 puncta on dTGN was quantified from 100 cells (n = 3, mean ± SD; two-sided t test). N.D., not detectable. **c**, Neither imiquimod- nor CL097-induced NLRP3 puncta were colocalized with mitochondria in ASC-deficient BMDMs. Cells were primed with LPS (50 ng/mL) for 3 hours and incubated −/+ imiquimod or CL097 (45 μg/mL) for 60 min, before immunostained for endogenous NLRP3 and TOM20 (mitochondrial marker). **d**, High extracellular KCl had no significant effect on imiquimod- or CL097-induced NLRP3 aggregation on dTGN. HeLa NLRP3-GFP cells were treated −/+ imiquimod or CL097 (45 μg/mL) in the presence of KCl (0 or 30 mM) for 80 min before imaging. Results were analyzed as in (**b**). n.s., not significant (alpha = 0.01).

**Figure 6.**
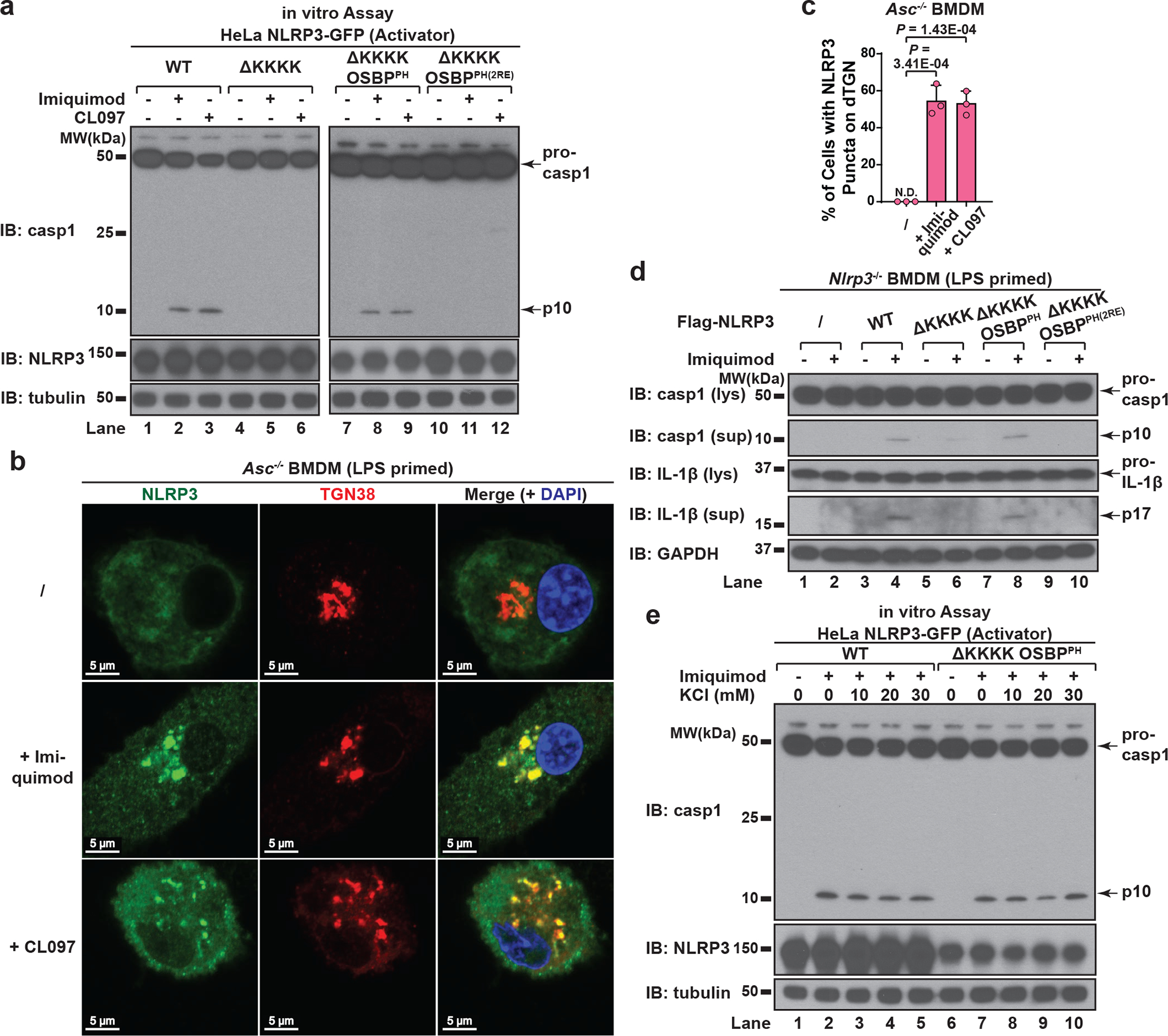
Binding to PI4P on dTGN is essential for K^+^ efflux-independent NLRP3 activation. **a**, HeLa cells stably expressing the indicated proteins were treated −/+ imiquimod or CL097 (45 μg/mL) for 80 minutes before cell lysates were analyzed by the in vitro NLRP3 activity assay. **b**, **c**, Primary ASC-deficient BMDMs were primed with LPS and incubated −/+ imiquimod or CL097 (45 μg/mL) for 60 minutes. The percentage of cells with NLRP3 puncta on dTGN was quantified from 100 cells (n = 3, mean ± SD, two-sided t test). N.D., not detectable. **d**, Primary NLRP3-deficient BMDMs reconstituted with the indicated proteins were primed with LPS and treated −/+ imiquimod. Cell lysates (lys) and supernatants (sup) were analyzed by immunoblotting with the indicated antibodies to assess the inflammasome activation. **e**, HeLa cells stably expressing the indicated NLRP3 proteins were treated −/+ imiquimod in the presence of increasing concentrations of KCl and the cell lysates were analyzed by the in vitro NLRP3 activation assay.

Recent studies have shown that NLRP3 can be activated by the immune modulators imiquimod and CL097 in a K^+^ efflux-independent manner^21,22^. Interestingly, we found that both imiquimod and CL097 also induced dramatic dispersion of TGN and recruitment of NLRP3 to dTGN. Moreover, NLRP3 puncta formation was blocked by deleting the KKKK motif, which could be restored by the replacement with OSBP^PH^ but not OSBP^PH(R107/108E)^ (Extended Data Fig. 9a–b). Importantly, both imiquimod- and CL097-mediated NLRP3 activation was abolished when the KKKK motif of NLRP3 was deleted, and could be rescued by the insertion of OSBP^PH^ but not OSBP^PH(R107/108E)^ (Fig. 6a). Similarly, endogenous NLRP3 was recruited to dTGN (Fig. 6b–c) but not mitochondria (Extended Data Fig. 9c) after imiquimod or CL097 treatment in primary ASC-deficient BMDMs. Moreover, the KKKK motif is essential for NLRP3 to restore caspase-1 and IL-1β cleavage in primary NLRP3-deficient BMDMs in response to imiquimod, and the KKKK motif could be replaced by OSBP^PH^ but not OSBP^PH(R107/108E)^ (Fig. 6d and Extended Data Fig. 8d). Thus, PI4P-mediated recruitment to dTGN is also essential for K^+^ efflux-independent NLRP3 activation.

Consistent with the fact that imiquimod and CL097 are K^+^ efflux-independent stimuli^22^, the recruitment of NLRP3 to dTGN (Extended Data Fig. 9d) and the activation of both WT NLRP3 and NLRP3(ΔKKKK OSBP^PH^) (Fig. 6e) were highly resistant to extracellular KCl. It’s unclear what contributes to the differences regarding the requirement of K^+^ efflux for different NLRP3 stimuli. One possibility is that K^+^ efflux-independent stimuli induced much more dramatic dispersion of TGN and partial separation of PI4P from other TGN compartments such as those marked by TGN38 (Extended Data Fig. 9a, see Supplementary Information), which may help expose PI4P in a more efficient conformation to recruit NLRP3 even without the induction of K^+^ efflux.

**Extended Data Figure 10.**
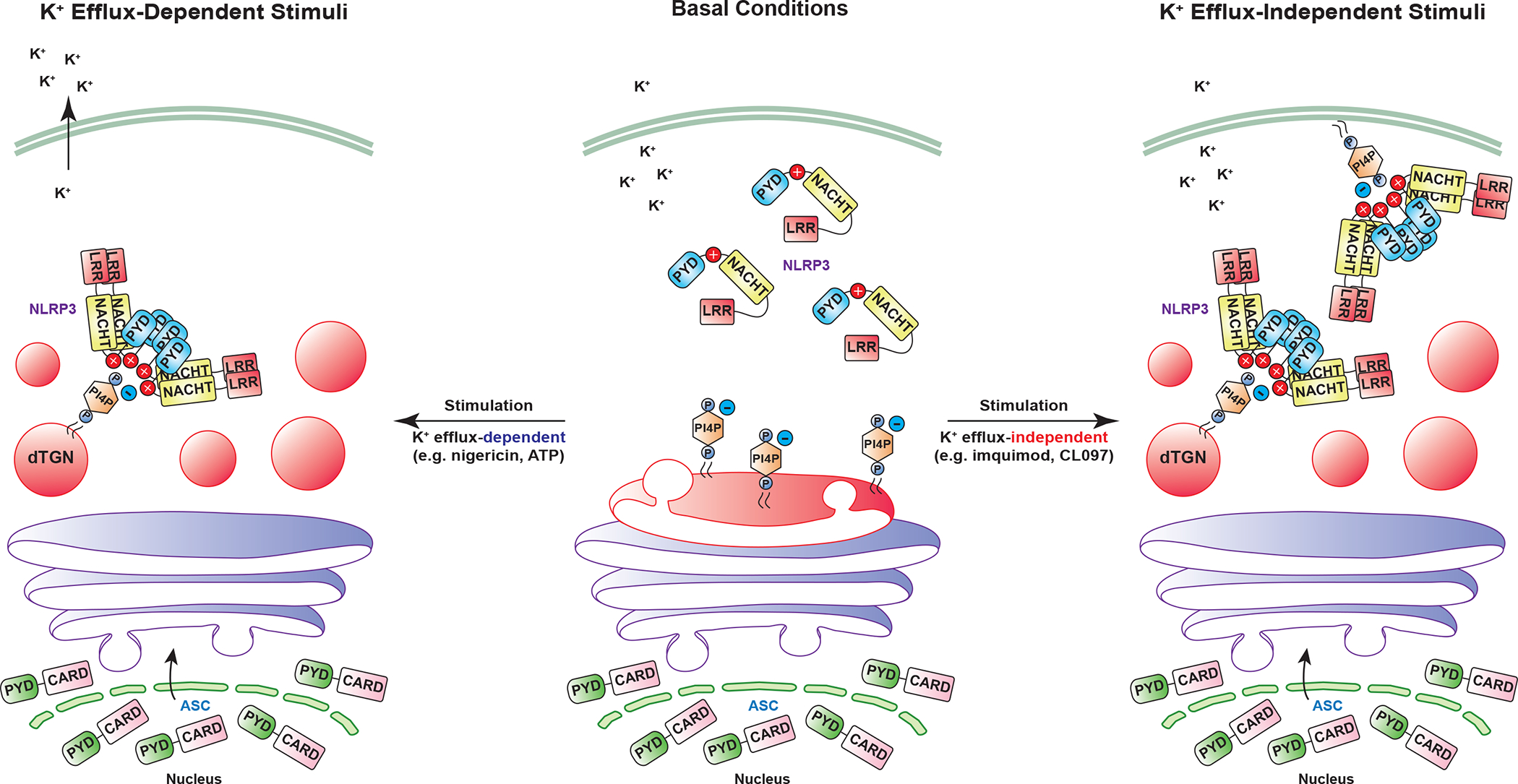
Model: NLRP3 aggregation on dTGN through PI4P binding is a common cellular signal essential for the inflammasome activation by diverse stimuli. Central panel: under basal conditions, NLRP3 is diffused in the cytosol while TGN (shown in red on the Golgi stack) remains as a single cluster attached to medial- and cis-Golgi (colored in purple). Left panel: when cells are stimulated with K^+^ efflux-dependent stimuli such as nigericin and ATP, TGN is disassembled into multiple dispersed structures (dTGN), whereas cis- and medial- Golgi stacks still remain intact. These stimuli also trigger K^+^ efflux, which helps recruit NLRP3 to dTGN via ionic bonding between the negatively-charged PI4P on dTGN membranes and the positively-charged polybasic region of NLRP3. Right panel: when cells are stimulated with K^+^ efflux-independent stimuli such as imiquimod and CL097, TGN is also disassembled, although in a more dramatic way. PI4P is partially separated from other TGN compartments and some can be enriched in the plasma membrane. NLRP3 is then recruited to these PI4P-containing microdomains through its polybasic region. For both types of stimulation, dTGN serves as a scaffold for NLRP3 to aggregate in the form of multiple puncta, which then interact with ASC to activate the downstream signaling cascade. This model shows that aggregation of NLRP3 on dTGN through PI4P binding is a common cellular signal essential for its activation by diverse stimuli.

In summary, using various complementary techniques in both reconstituted systems and primary macrophages, we have unveiled a unique common cellular signal triggered by diverse NLRP3 stimuli: the disassembly of TGN into dTGN, to which NLRP3 is recruited via ionic bonding between its polybasic region and PI4P on dTGN (Extended Data Fig. 10). dTGN then serves as a scaffold for NLRP3 to aggregate and interact with ASC to activate the downstream cascade. This mechanism of NLRP3 activation is reminiscent of the “guard model” in plants, in which the disease resistant (R) proteins indirectly recognize virulent factors by monitoring the integrity of host targets, so-called “pathogen-induced altered self”^23,24^. By binding to dTGN as the “altered self”, NLRP3 indirectly senses a large variety of pathogen- and danger-associated molecules.

## METHODS

### Antibodies and Chemicals

Antibodies against NLRP3 (AG-20B-0014) and ASC (AG-25B-0006) were from AdipoGen. Antibodies against caspase-1 (sc-515), human TGN38 (sc-33783) and TOM20 (sc-11415) were from Santa Cruz Biotechnology. Antibody against murine TGN38 was a kind gift from Dr. Matthew Seaman (Cambridge Institute for Medical Research). Antibodies against GOLGA4 (611280) and GM130 (610822) were from BD Biosciences. Antibodies against tubulin (T5168), Flag (F1804) and AP1G1 (A4200) were from Sigma. Antibody against HA (mms-101p) was from Covance. Antibody against giantin (ab24586) was from Abcam. Antibody against COX IV (20E8C12) was from Invitrogen. Antibodies against calreticulin (#2891) and GAPDH (#2118) were from Cell Signaling. Antibody against IL-1β (AB-401-NA) was from R&D Systems. Antibody against ERGIC-53 (ALX-804-602-C100) was from Axxora. Alexa Fluor secondary antibodies (488, 568, and 633) were from Life Technologies.

Flag antibody-conjugated agarose (A2220), nigericin (N7143) and ATP (A7699) were from Sigma. Gramicidin (ALX-350-233) was from Enzo life sciences. Rapamycin (AG-CN2-0025) was from AdipoGen. LPS (Ultra-Pure), imiquimod (tlrl-imq) and CL097 (tlrl-c97-5) were from InvivoGen.

### Mammalian Cell Culture

Cell lines including HEK293T, HeLa, COS-7 and RAW 264.7 were cultured in Dulbecco’s modified Eagle’s medium (DMEM) supplemented with 10% (v/v) cosmic calf serum (Hyclone), penicillin (100 U/ml) and streptomycin (100 μg/ml). These cell lines were originally obtained from ATCC (https://www.atcc.org/), and were constantly monitored for contamination from other cell lines. They were free of mycoplasma contamination based on the results of e-Myco Mycoplasma PCR Detection Kit (Bulldog Bio) and were regularly maintained with Normocin (antimicrobial reagent against mycoplasma, bacteria and fungi) (InvivoGen).

For primary BMDM induction, cells were isolated from mouse bone marrow and cultured in mCSF-1-containing RPMI 1640 medium supplemented with fetal bovine serum, penicillin and streptomycin as mentioned above. All cells were cultured at 37°C in an atmosphere of 5% (v/v) CO_2_. NLRP3-deficient mice (B6.129S6-*Nlrp3*^*tm1Bhk*^/J) were provided by Dr. Bruce Beutler (UT Southwestern Medical Center), and were originally from The Jackson Laboratory (Stock No: 021302). Bones from ASC-deficient mice were provided by Dr. Chandrashekhar Pasare (UT Southwestern Medical Center). All mice were bred and maintained under specific pathogen-free conditions in the animal care facility of University of Texas Southwestern Medical Center according to experimental protocols approved by the Institutional Animal Care and Use Committee.

### Inflammasome Stimulation

For stimulation of reconstituted cell lines, the culture medium was replaced by OPTI-MEM medium (Life Technologies) containing inflammasome stimulus including nigericin (10 μM), gramicidin (5 μM), ATP (5 mM, pH adjusted to 7.5), imiquimod (45 μg/mL) or CL097 (45 μg/mL). The cells were then incubated at 37 °C in an atmosphere of 5% (v/v) CO_2_ for 60 min (HEK293T) or 80 min (HeLa, COS-7 and BJ) unless otherwise specified. Poly(dA:dT) (Sigma) was transfected with Lipofectamine 2000 (Invitrogen) at 1.5 μg/mL for 3 hours. Priming with TLR ligands is not required for reconstituted cells lines due to the high expression level of stably introduced NLRP3, in line with previous studies^25,26^.

For stimulation of primary BMDM or RAW 264.7, the cells were primed with LPS (50 ng/mL) for 3 hours before NLRP3 stimulus was added: nigericin (10 μM), ATP (5 mM, pH adjusted to 7.5), imiquimod (45 μg/mL) or CL097 (45 μg/mL) for 60 min unless otherwise specified.

Cells were harvested in Lysis Buffer A [20 mM Tris·HCl (pH 7.5), 150 mM NaCl, 0.5% NP-40, 1 mM DTT, protease inhibitor cocktail (Roche)] and centrifuged at 20,000 g for 15 min to collect lysate for immunoblotting. For primary BMDM and RAW 264.7, the medium (supernatant) was also collected for detection of secreted caspase-1 p10 and IL-1β p17.

### Plasmids, Viruses and Stable Cell Lines

The lentiviral vectors for expressing proteins, pTY-EF1a-puroR/hygroR/zeoR-2A-GFP-Flag, were modified from a vector kindly provided by Dr. Yi Zhang (University of North Carolina at Chapel Hill). These vectors were further modified into pTY-EF1a-GFP-IRES- puroR/hygroR/zeoR, to circumvent the problem of incomplete cleavage by the 2A protease.

cDNA encoding genes were purchased from Life Technologies or Open Biosystems, and cloned into the lentiviral vectors described above. NLRP3, ASC and caspase-1 genes were of mouse origin, with the exception of human NLRP3 used in Extended Data Fig. 1e. For NLRP3(4KA)-GFP-GOLGA4^GRIP^, the C-terminal 312 amino acids of mouse GOLGA4 gene was fused to the C-terminus of NLRP3(K127/128/129/130A)-GFP separated by a short linker (RSIAT). All the other genes used in this study are of human origin unless otherwise specified. HomoloGene database (NCBI) was used to search for all currently identified NLRP3 orthologs and the results were further confirmed by Protein BLAST.

OSBP^PH^-GFP consists of Met-OSBP PH domain (human, amino acids 87–185) followed by a short linker (RSIAT) and GFP. For NLRP3(ΔKKKK), NLRP3 (ΔKKKK OSBP^PH^) and NLRP3 (ΔKKKK OSBP^PH(R107/108E)^), a short linker (GGGGS) was inserted at the position of deleted amino acids 127–130 to maintain the structural flexibility between different domains. ASC^PYD^ consists of only the pyrin domain of murine ASC (amino acids 1–90).

MitoDsRed2 protein was stably expressed in HeLa NLRP3-GFP cells by infection of lentiviral vector pCDH-DsRed2-Mito. The DsRed2-Mito gene in this lentiviral vector was cloned from pDsRed2-Mito (Clontech, #632421), which consists of DsRed2 fused with the mitochondrial targeting sequence of COX VIII at its N-terminus. The plasmid containing the coding sequence of PFO from the bacterium *Clostridium perfringens* was kindly provided by Dr. Russell DeBose-Boyd (UT Southwestern Medical Center).

With the exception of the inducible phosphatase recruitment system, all proteins were stably expressed through lentiviral infection, which was performed as described previously^27^. Briefly, lentivirus was packaged by co-transfecting HEK293T cells with the lentiviral vector and packaging vectors. Medium containing lentivirus was filtered and added to target cells in the presence of polybrene (10 μg/ml). Cells were then selected with respective antibiotics for at least seven days before protein expression was confirmed by immunoblotting and fluorescence microscopy.

### In Vitro Assay for NLRP3 Activation

As shown in the schematic of Fig. 1a, two HEK293T stable cell lines were set up that stably expressed only NLRP3 (293 NLRP3) or only ASC and caspase-1 (293 ASC + casp1). 293 NLRP3 was incubated with or without inflammasome stimulus (e.g. nigericin), before the cells were harvested in Lysis Buffer B [10 mM Tris·HCl (pH 7.5), 10 mM KCl, 1.5 mM MgCl_2_, 0.2% NP-40, protease inhibitor cocktail] and centrifuged at 1,000 g for 5 min. 10 μg of supernatant (cell extracts) from 293 NLRP3 (“Activator”) was then mixed with 10^6^ cells from 293 ASC + casp1 (“Recipient”) semi-permeabilized with 60 ng PFO and additional Lysis Buffer B was added to reach a final volume of 10 μL. After incubation at 30 °C for 80 min, the reaction mixture was incubated with additional 0.5% NP-40 on ice for 15 min before centrifugation at 20,000g for 15 min. The supernatant was boiled in SDS loading buffer and used for caspase-1 immunoblotting. In later experiments, other cells that expressed NLRP3 but not ASC (e.g. HeLa NLRP3-GFP and RAW 264.7) were also used as ‘Activator’ cells. In addition, ASC and caspase-1 in Recipient cells can be replaced by the fusion protein PYD-p20-p10 consisting of pyrin domain (PYD, amino acids 1–104) from ASC and the C-terminal p20-p10 region from caspase-1 (amino acids 92–402), a fusion protein reported to bypass the CARD-CARD interaction between ASC and caspase-1^28^.

### Fractionation of HEK293T NLRP3-GFP Cells and Purification of NLRP3

20 plates (15 cm in diameter) of HEK293T cells stably expressing Flag-NLRP3-GFP were incubated with or without nigericin treatment, homogenized in Isotonic Buffer [0.25 M sucrose, 10 mM Tris·HCl (pH 7.5), 10 mM KCl, 1.5 mM MgCl_2_ and protease inhibitor cocktail] and centrifuged at 1,000 g for 5 min to remove nucleus pellet (P1). The supernatant (S1) was further centrifuged at 5,000 g for 10 minutes to get heavy membrane fraction (pellet, P5), while the supernatant (S5) was centrifuged at 100,000 g for 20 min to separate light membrane fraction (pellet, P100) from cytosol fraction (supernatant, S100). P5 and P100 were washed with Isotonic Buffer once and resuspended in the same buffer.

P5 and P100 fractions were then used for sucrose gradient ultracentrifugation separately. Briefly, sucrose solutions in Low Salt Buffer [10 mM Tris·HCl (pH 7.5), 10 mM KCl, 1.5 mM MgCl_2_] were loaded in a centrifuge tube (from bottom to top, 60%, 50%, 40%, 30%, 20%, 2.1 mL/layer). 0.8 mg P5 or P100 was loaded on top of the gradient and centrifuged at 170,000 g for 2 hours. 9 fractions (1.2 mL/fraction) were collected from top to bottom. For each fraction, 3 μL (for P5) or 1uL (for P100) was used for the in vitro activity assay while 10 μL was used for immunoblotting against various proteins.

Fraction #4 (from top) of P5 sucrose gradient was used for further purification. Fraction #4 was first incubated with 0.2% NP-40 on ice for 20 minutes before centrifuged at 5,000 g for 10 minutes. The supernatant (Fraction Extract) was immunoprecipitated with Flag M2 agarose in the presence of additional KAc (110 mM) and NaCl (100 mM) at 4 °C for 6 hours. The agarose beads were then washed for five times with Flag IP Wash Buffer A [10 mM Tris·HCl (pH 7.5), 10 mM KCl, 1.5 mM MgCl_2_, 110 mM KAc, 100 mM NaCl, 0.05% NP-40] before eluted in Low Salt Buffer with Flag peptides overnight. The elute was filtered and concentrated using 10 kDa cutoff concentrator before the purity of NLRP3 was confirmed by both silver staining and mass spectrometry.

### Enrichment of Nigericin-Induced NLRP3 Puncta

293 NLRP3-GFP cells were incubated with or without nigericin for 60 min, before washed with PBS twice and incubated with or without PBS containing 0.3% saponin for 10 min. The cells (still attached to plates) were then washed with PBS again and imaged by fluorescence microscopy. Afterwards the cells were collected and lysed with Lysis Buffer B, before the cell extracts were used as “Activator” for the in vitro assay.

### Functional Rescue of Primary NLRP3-Deficient BMDM

Bone marrow cells were collected from NLRP3-deficient mice and cultured in mCSF-1-containing medium. On day 2 and day 3 respectively, cells were infected with lentivirus encoding pTY-EF1a-zeoR-2A-Flag-NLRP3(WT or mutants). As a control, equal volume of medium without lentivirus was added to cells. On day 7 post-induction in mCSF-1-containing medium, cells were primed with LPS (50 ng/mL) for 3 hours (to induce pro-IL-1β) before nigericin (10 μM) or imiquimod (45 μg/mL) was added for another 60 min. The cell lysate and medium (supernatant) were then collected for immunoblotting.

### K^+^ Efflux Inhibition and Induction

To inhibit nigericin-induced K^+^ efflux, additional KCl was added into OPTI-MEM medium at the indicated final concentrations at the same time with nigericin. Up to 30 mM of extracellular KCl has been reported to specifically inhibit NLRP3 inflammasome but not other inflammasome pathways or general cellular signaling^29^.

To induce spontaneous K^+^ efflux without adding NLRP3 stimuli, cells were incubated in K^+^-Free Hanks Buffer [145 mM NaCl, 1.3 mM CaCl_2_, 1.0 mM MgSO_4_, 10 mM HEPES (pH 7.5), 5.5 mM glucose] for the indicated time period. As a control, cells were incubated in Regular Hanks Buffer [140 mM NaCl, 5 mM KCl, 1.3 mM CaCl_2_, 1.0 mM MgSO_4_, 10 mM HEPES (pH 7.5), 5.5 mM glucose] with or without nigericin (10 μM) for the similar time length.

### Measurement of Intracellular K^+^ Concentration

Cells were washed with ice-cold PBS and centrifuged at 1,000 g for 5 min to remove residual buffer; this process was repeated once. The cell pellets were then resuspended in Milli-Q H_2_O at a volume equal to cell pellet volume, before snap-frozen with liquid nitrogen and thawed repeatedly for a total of six cycles to lyse the cells. The lysed cells were centrifuged at 14,000 g for 10 minutes and the cell extracts (supernatant) were collected. The volumes of cell extracts were further adjusted according to protein concentrations, before measured by Jenway Flame Photometer (PFP7) for K^+^ concentration, with the help from Dr. Michel Baum and Susan Legan in O’Brien Kidney Research Center (UT Southwestern Medical Center). A standard curve with titrated KCl solutions was made for each experiment to ensure the results fell within the linear range of measurement.

### Immunostaining and Fluorescence Microscopy

For immunostaining, cells were fixed with 4% paraformaldehyde and permeabilized with 0.1% Triton X-100 in PBS, before incubated with primary antibodies followed by Alexa Fluor secondary antibodies, while nuclei were stained with DAPI in the mounting medium (Vectashield). Later we also used 0.1% saponin in place of 0.1% Triton X-100 in permeabilization step to better preserve the dTGN structures in fixed cells. For immunostaining of endogenous NLRP3 in primary BMDM, Tyramide Signal Amplification Kits (T20948 and T20949, Life Technologies) were used to enhance the signal-to-noise ratio. For imaging with multiple channels, extensive controls were performed to make sure there was no nonspecific staining or crosstalk between channels. These controls include: a) use cells that lack one of the proteins of interest; and/or b) perform staining without one of the primary antibodies or secondary antibodies.

Fluorescence images for fixed cells were taken with Zeiss LSM 700 confocal laser scanning microscope. Time-lapse imaging of live cells was performed using Nikon A1R microscope equipped with Tokai Hit Incubator System. Phase contrast and fluorescence imaging of live cells were taken with EVOS FL Cell Imaging System (Thermo Fisher Scientific).

### Transmission Electron Microscopy

HeLa cells were incubated with or without nigericin for 80 min before fixed with 2.5% (v/v) glutaraldehyde in 0.1 M sodium cacodylate buffer. After rinsed with 0.1 M sodium cacodylate buffer for three times, the cells were post-fixed in 1% osmium tetroxide and 0.8% potassium ferricyanide in 0.1 M sodium cacodylate buffer for 1 hour. Cells were then rinsed with water and stained with 2% aqueous uranyl acetate overnight. After rinsing with water for three times, cells were dehydrated with increasing concentrations of ethanol, infiltrated with Embed-812 resin and polymerized in a 60 °C oven overnight. Blocks were sectioned with a diamond knife (Diatome) on a Leica Ultracut UC7 ultramicrotome (Leica Microsystems) and collected onto copper grids, before post-stained with 2% Uranyl acetate in water and lead citrate. Images were acquired on a Tecnai G2 spirit transmission electron microscope (FEI) equipped with a LaB6 source using a voltage of 120 kV with the help from Electron Microscopy Core Facility (UT Southwestern Medical Center).

### PIP Strip Assay

The following proteins were purified from HEK293T stable cell lines: Flag-GFP, Flag-NLRP3(127–146)-GFP, and Flag-NLRP3(127–146, K127/128/129/130A)-GFP. The C-terminal GFP tag was introduced to increase the stability of short fragments expressed in mammalian cells. These cells were lysed in Lysis Buffer C [10 mM Tris·HCl (pH 7.5), 10 mM KCl, 1.5 mM MgCl_2_, 500 mM NaCl, 0.2% NP-40, and protease inhibitor cocktail] and centrifuged at 20,000 g for 15 min. The supernatant (lysate) was used for Flag immunoprecipitation (IP) with M2 beads at 4 °C for 6 hours. The beads were then washed in Flag IP Wash Buffer B [10 mM Tris·HCl (pH 7.5), 10 mM KCl, 1.5 mM MgCl_2_, 500 mM NaCl, 0.1% Tween20] for five times before eluted with the same buffer containing Flag peptide. The elute was filtered and concentrated using 10 kDa cutoff concentrator, before aliquots were used for Coomassie Blue staining and PIP Strip binding assay.

The binding assay was performed with PIP Strip (P-6001) (Echelon Biosciences) according to the protocol from the manufacturer. Briefly, PIP Strip membrane was first blocked with PIP Strip Block Buffer [PBS with 0.1% Tween-20 and 3% fat-free BSA] for 1 hour, before incubated with 2.5 μg of purified target protein in fresh PIP Strip Block Buffer for another hour. The membrane was then washed with PIP Strip Wash Buffer [PBS with 0.1% Tween-20] for three times (10 minutes each) and incubated with PIP Strip Wash Buffer containing Flag M2 antibody for 1 hour. After that the membrane was washed for three times before incubation with HRP mouse antibody in PIP Strip Wash Buffer for another hour. All the steps above were performed at room temperature. The PIP Strip membranes were then stained similar to regular immunoblotting. All samples were examined at the same time with similar exposure time. The experiment has been repeated in a total of three times with similar results.

### Inducible Recruitment of Phospholipid Phosphatases

Plasmids pTGN38-FRB-CFP and pmRFP-FKBP12-Sac1 (human, amino acids 2–516, with transmembrane domain removed) were kindly provided by Dr. Tamas Balla (National Institutes of Health), while pmCherry-FKBP12-MTM1 (human) was ordered from Addgene (deposited by Tamas Balla Lab). We also replaced Sac1 gene in pmRFP-FKBP12-Sac1 with other phosphatase genes to make pmRFP-FKBP12-Sac2 (human), pmRFP-FKBP12-lipin1(human) and pmRFP-FKBP12-Fig4(human).

Briefly, we set up a COS-7 cell line stably expressing Flag-NLRP3. A single colony was selected to ensure homogenous expression of Flag-NLRP3 in all cells, and its behaviors (including stimulus-triggered dTGN formation, NLRP3 recruitment and activation) were confirmed to be similar to the original pooled cells. This stable cell line was then transiently transfected with both pTGN38-FRB-CFP and pmRFP(or mCherry)-FKBP12-phosphatase vectors (1 μg/well of 6-well plate for each vector) with Lipofectamine 2000 (Thermo Fisher Scientific) in OPTI-MEM medium. The medium was changed to regular medium 8 hours post transfection, and the cells were allowed to recover for another 2 hours, before split onto slides. After overnight culture, the cells were treated with rapamycin (1 μM) in the absence or presence of nigericin (10 μM) for 80 min before immunostained with Flag antibody and Alexa Fluor 633 (pseudocolored to green).

### Statistics and Reproducibility

Representative results from at least three experiments are shown for every figure except otherwise specified in the figure legends.

For quantification of percentages of cells with the phenotype of interest, 25 non-overlapping whole-field images were randomly taken throughout the slide of each sample. Only DAPI channel was used during the random selection of whole-field images to avoid bias in selection of cells with particular phenotypes. The number of those with phenotype of interest was recorded from 100 cells and this process was repeated for a total of three times. For quantification of TGN disassembly during the early time points (the first 30 minutes) of stimulation in primary WT BMDM, 20 non-overlapping whole-field images were randomly taken throughout the slide of each sample and the number of TGN structures not connected with each other was quantified for 100 cells and grouped. Cells at mitosis stage were excluded for the quantifications above based on chromosomes morphology in DAPI channel, because these cells had mitotic Golgi disassembly. Data are presented as mean ± SD. Statistical analysis was performed using t test (two-sided) in GraphPad Prism 7. Statistical significance was determined with the Holm-Sidak method, with alpha = 0.01. Intracellular K^+^ measurement was statistically analyzed with the same t test method. For colocalization analysis, images were selected similar to methods above, before Pearson’s correlation coefficient (threshold regression: Costes) was calculated using Coloc 2 plugin of ImageJ (version 1.51n)^30^.

## Extended Data

## Supplementary Material

Supplementary Material

supplementary video 1

Supplementary video 2

Supplementary video 3

Supplementary video 4

## Data Availability

All important data generated or analyzed during this study are included in this article. Additional supplementary data are available from the corresponding author upon request.
