# Supplementary material for: PtdIns4P on Dispersed Trans-Golgi Network Mediates NLRP3 Inflammasome Activation
Source: Nature. Author manuscript; Available in PMC 2022 Aug 25. (PMC9402428; doi:10.1038/s41586-018-0761-3)
Supplement: Supplementary Material [file NIHMS1829173-supplement-Supplementary_Material.pdf]

Supplementary Results

Dispersed-TGN-localized puncta are the active form of NLRP3

To examine whether the stimulus-triggered puncta represent the active form of NLRP3, we incubated 293 NLRP3-GFP cells with saponin to enrich the puncta form of NLRP3. Saponin is a type of amphipathic glycoside that generates small holes on plasma membrane but leaves subcellular organelles largely intact[1]. After washing with PBS, cytosolic NLRP3 was largely depleted, while nigericin-induced NLRP3 puncta still retained inside cells (Extended Data Fig. 1g, left panel). This puncta-enriched sample had much higher signal-dependent activity compared to cell extracts collected with regular methods (Extended Data Fig. 1g, right panel; compare lane 4 and 6). To further explore whether NLRP3 needs aggregation in order to become active, we examined NLRP3 mutations that have been shown to cause auto-inflammatory diseases in both humans and mice[2,3]. As shown in Extended Data Fig. 1h, while wild type (WT) NLRP3 formed puncta only when cells were stimulated with nigericin, all three constitutively active mutants (R258W, A350V and L351P) of NLRP3 aggregated into various puncta without stimulation, again suggesting that NLRP3 forms multiple puncta to become active.

To further confirm that dispersed TGN (dTGN)-localized NLRP3 puncta are the active form of NLRP3, we tested whether these puncta were able to polymerize ASC. In the presence of full-length ASC, NLRP3 puncta were rapidly incorporated into a single speck with ASC (Extended Data Fig. 1f) due to the prion-like property of ASC[4,5], making it difficult to study the intermediate state of ASC polymerization from NLRP3 puncta. Therefore, we focused on ASC-PYD (consisting of only the pyrin domain of ASC, amino

acids 1-90), which forms multiple long filaments instead of a single speck after stimulation[4,6,7], and therefore is a good tool to study whether ASC aggregation initiates from NLRP3 puncta. Strikingly, nigericin treatment induced ASC-PYD to form elongated filaments extending from NLRP3 puncta localized on dTGN (Extended Data Fig. 4d). These results thus indicate that dTGN-localized puncta are the active form of NLRP3.

TGN dispersion induced by NLRP3 stimuli is a highly specific cellular process

The complete disassembly of TGN has been confirmed by immunostaining against various TGN-localized proteins, including but not limited to TGN38, GOLGA4 and AP1G1 (Fig. 2a, Extended Data Fig. 2c and 7g). Interestingly, there were at least two distinct populations of nigericin-induced dTGN vesicles marked by the presence of TGN38 and GOLGA4 respectively, and both groups of vesicles were able to recruit NLRP3 (Extended Data Fig. 2c).

The disassembly of TGN induced by NLRP3 stimuli is highly specific, because the same stimuli did not cause disassembly of the closely associated cis- and medial-Golgi (Extended Data Fig. 2d-e). A recent study[8] has proposed that cis/medial-Golgi is involved in NLRP3 inflammasome activation. This is different from our conclusion that dispersed TGN plays a critical role in NLRP3 recruitment and activation. The authors showed that in the presence of ASC, the single NLRP3-ASC speck was located close to (but not overlapped with) the still intact giantin-marked cis/medial-Golgi. Our closer analysis

revealed that the NLRP3-ASC speck is localized on dispersed TGN (Extended Data Fig. 4d).

Further imaging studies showed no detectable morphological change in other organelles, including mitochondria (Extended Data Figure 4a-b and 9c), ER, ER-Golgi intermediate compartment (ERGIC), COPI vesicles, late endosome, peroxisome, and centrosome (data not shown). However, the early endosome marker EEA1 could be detected on some but not all of dTGN structures (Extended Data Fig. 2f), probably due to the constant cargo transfer between TGN and early endosomes. In addition, HeLa cells (without the intact inflammasome pathway, and therefore no pyroptosis) treated with nigericin were generally healthy except the appearance of giant dTGN vesicles as shown by bright-field images (Extended Data Fig. 2a and 7e), transmission electron microscopy (Extended Data Fig. 2b) and lactate dehydrogenase (LDH) cytotoxicity assay (data not shown). Together, these results have shown that NLRP3 stimuli-induced TGN disassembly is a highly specific cellular event.

Endogenous NLRP3 is recruited to stimulus-induced dTGN in physiologically relevant cells

To confirm that signal-dependent dTGN formation occurs in physiologically relevant cells, we stimulated LPS-primed primary WT bone marrow-derived macrophages (BMDMs) with either nigericin or ATP for a short time course spanning 30 minutes (to prevent the interference by pyroptosis occurring later). Dramatic TGN disassembly could be detected as early as 5 and 10 minutes post ATP and nigericin treatment respectively

(Extended Data Fig. 3a), which preceded the earliest detectable caspase-1 and IL-1β cleavage (Extended Data Fig. 3b-c).

Importantly, when ASC-deficient primary BMDMs were treated with nigericin or ATP, endogenous NLRP3 aggregation on dTGN could be observed in more than 20% of cells at 10 minutes post nigericin stimulation (Extended Data Fig. 3d), which occurred earlier than caspase-1 and IL-1β cleavage in WT BMDMs (Extended Data Fig. 3b). Note that ASC-deficient BMDMs instead of WT BMDMs were used for imaging of the dTGN recruitment of endogenous NLRP3, because it is difficult to detect NLRP3 puncta formation in cells that express ASC. Due to the prion-like property of ASC[4,5], the first NLRP3-ASC speck assembles at such a high speed that it dramatically decreases the concentration of soluble ASC below a certain threshold, thus preventing the formation of new specks. This is consistent with previous observation that ASC aggregation is an energetically favorable reaction, resulting in "all or none" formation of ASC speck[9]. Indeed, this may be a reason why the formation of multiple NLRP3 puncta on dTGN had remained unnoticed until now, and it's the use of our reconstitution systems and cells lacking ASC that allows us to detect this important cellular event.

AIM2 activation doesn't require dTGN recruitment

To reconstitute the step of AIM2 activation, we stably expressed AIM2 in HeLa cells and transfected the cells with poly(dA:dT), a synthetic DNA polymer that specifically activates the AIM2 pathway. This led to AIM2 aggregation around DNA dots in the cytosol as reported previously[10,11], but didn’t trigger TGN dispersion or AIM2

recruitment to TGN (Extended Data Fig. 2i). This is consistent with the result that poly(dA:dT)-induced AIM2 aggregation was not affected by depletion of PtdIns4P on TGN (Extended Data Fig. 7f). These results suggest that dTGN formation and recruitment of an upstream sensor do not occur in all inflammasome pathways.

The second positively-charged region of NLRP3 is also important for its dTGN recruitment and activation

Besides the KKKK motif, we also identified a second positively charged region located after the KKKK motif, spanning amino acids 134 to 143. This region consists of five positively charged residues (lysine or arginine) that are highly conserved in all currently known NLRP3 orthologs (Fig. 3b). Mutations of these residues to alanine impaired dTGN recruitment (Extended Data Fig. 6f) and activation (Extended Data Fig. 6g) of NLRP3 in a manner dependent on the number of the remaining positively charged residues, suggesting that this region also plays a critical role in mediating NLRP3 aggregation on dTGN and its subsequent activation, together with the KKKK motif. This likely explains the residual dTGN recruitment of NLRP3 mutants 4KA (K127/128/129/130A) (e.g. Fig. 3c) and ΔKKKK (e.g. Extended Data Fig. 8a), for which the second positively-charged region could partially compensate.

PtdIns4P is the phospholipid required for NLRP3 recruitment and activation

To examine which phospholipid is responsible for recruitment of NLRP3 to dTGN in live cells, we used the recently developed inducible recruitment system of phospholipid phosphatases[12]. Basically, COS-7 cells stably expressing full-length Flag-NLRP3 were transiently transfected with different phosphatases fused with FKBP12, along with TGN38 fused with the FKBP12-rapamycin binding (FRB) domain of mTOR. Upon addition of rapamycin, FRB forms a heterodimer with FKBP12, thus recruiting the phosphatase to TGN where it hydrolyzes its target phospholipid (Fig. 4a). Without rapamycin, Sac1 (a PtdIns4P phosphatase) fused with FKBP12 was located throughout the whole cell, and had no effect on nigericin-induced NLRP3 puncta formation. After rapamycin treatment, FKBP12-Sac1 was translocated to TGN and blocked NLRP3 aggregation (Fig. 4b). In contrast, TGN translocation of the catalytically inactive mutant (C389S) of Sac1 didn’t affect NLRP3 recruitment (Extended Data Fig. 7b). Similar to Sac1, another highly specific PtdIns4P phosphatase Sac2[13] also abolished NLRP3 recruitment, whereas phosphatases that target other phospholipids, including lipin1 (for phosphatidic acid (PA)), Fig4 (for PtdIns(3,5)P2) and MTM1 (for PtdIns3P and PtdIns(3,5)P2), had no detectable effect on NLRP3 recruitment (Extended Data Fig. 7c). The reason that NLRP3 showed higher specificity in lipid biding in live cells compared to the in vitro blot assay is likely because of the usage of pure lipids immortalized on nitrocellulose membranes in lipid blot assay, while physiologically-relevant membranes only contain at most 1% of the phospholipid of interest[14]; moreover, the presence of membrane-associated proteins and the biophysical features of membranes (e.g. high curvature of dTGN) may also contribute to the specificity of protein-lipid interactions[15].

Note that neither dTGN recruitment (Fig. 4c) nor activation (Fig. 4d) of NLRP3 was completely abolished by TGN-targeted Sac1 because the expression of this phosphatase has been controlled at a modest level (Extended Data Fig. 7d) to avoid causing general defects in cells, and therefore the PtdIns4P pool on TGN was significantly decreased but not completely depleted (data not shown).

As shown in Fig. 2a (inset images) and Supplementary Video 2, NLRP3 puncta were restricted to certain regions on dTGN, and therefore only shared limited colocalization with general TGN markers such as TGN38 (which were distributed relatively evenly on dTGN). This is because NLRP3 specifically aggregates on PtdIns4P-enriched microdomains, as evidenced by its strong colocalization with OSBP-PH, one of the best characterized PtdIns4P-binding domains[16], but not AP-1 complex (marked by AP1G1), which was also present on dTGN (Extended Data Fig. 7g) but mainly relied on ARF1 for TGN targeting[17,18].

The KKKK motif functions as a PtdIns4P-binding domain for NLRP3 recruitment and activation

Both NLRP3(ΔKKKK/OSBP-PH) and NLRP3(ΔKKKK/OSBP-PH(R107/108E)) were expressed at lower levels than WT NLRP3 and NLRP3(ΔKKKK) (e.g. Fig. 5a and 5e), probably due to their larger size (OSBP-PH consists of aa 87-185 from human OSBP while the KKKK motif only consists of four residues). To confirm that the inability of NLRP3(ΔKKKK/OSBP-PH(R107/108E)) to become active is not due to its lower protein level, we expressed this protein at comparable level with WT NLRP3 through titrations

of lentivirus encoding the proteins, and found that NLRP3(ΔKKKK/OSBP-PH(R107/108E)) still had no detectable activity while WT NLRP3 possessed strong signal-dependent activity (Extended Data Fig. 8c), thus confirming that the defect of this mutant is caused by its inability to be recruited to dTGN after stimulation.

To determine if recruitment to non-PtdIns4P-enriched regions on TGN is sufficient for NLRP3 activation, we fused NLRP3(4KA) to the C-terminal GRIP domain of GOLGA4, which binds to TGN in a PtdIns4P-independent manner[19]. This fusion protein was constitutively localized on TGN, but had no detectable activity upon nigericin stimulation, in contrast to NLRP3(ΔKKKK/OSBP-PH) (Extended Data Fig. 8e). This result indicates that targeting to PtdIns4P-enriched microdomains rather than general TGN localization is essential for the activation of NLRP3.

It remains to be studied how the dispersion of TGN by NLRP3 stimuli promotes NLRP3 recruitment and aggregation. One hypothesis is the formation of dTGN may help arrange PtdIns4P on the membrane in a different conformation that allows NLRP3 to aggregate. A small fraction of NLRP3 may be constantly in the equilibrium state that loosely binds to PtdIns4P on TGN under basal conditions, but it is only after TGN dispersion that NLRP3 can aggregate and further recruit other NLRP3 molecules to PtdIns4P microdomains, leading to its activation. This hypothesis is consistent with the observation that a small fraction of NLRP3 was present in membrane fractions (Fig. 1c) and co-migrated with TGN marker (Fig. 2f and Extended Data Fig. 4c) under basal conditions.

K^+ efflux alone is not sufficient for NLRP3 activation

Incubation of HeLa NLRP3-GFP cells in K^+-free medium for 80 minutes was sufficient to drive spontaneous K^+ efflux not weaker than that triggered by nigericin (Extended Data Fig. 8g), but failed to activate either WT NLRP3 or NLRP3(ΔKKKK/OSBP-PH) (Extended Data Fig. 8h). Similarly, incubation in K^+-free medium for 30 minutes induced spontaneous K^+ efflux not weaker than that induced by nigericin treatment in WT BMDMs (Extended Data Fig. 8i), but didn't trigger caspase-1 or IL-1β cleavage even with incubation up to 120 minutes (Extended Data Fig. 8j). These results indicate that K^+ efflux alone is not sufficient for NLRP3 activation. This is consistent with our data that nigericin stimulation is still required for NLRP3(ΔKKKK/OSBP-PH) to become active even though it no longer requires K^+ efflux (Fig. 5e). Moreover, for K^+ efflux-independent stimuli such as imiquimod, K^+ efflux is not required for the activation of either WT NLRP3 or NLRP3(ΔKKKK/OSBP-PH) (Fig. 6e). These data thus are consistent with the hypothesis that TGN dispersion (a K^+ efflux-independent cellular signal as shown in Fig. 5c) is important for NLRP3 recruitment and activation.

K^+ efflux-independent stimuli induced partial separation of PtdIns4P from other TGN compartments

We noticed that WT NLRP3 puncta induced by K^+ efflux-independent stimuli imiquimod and CL097 only partially colocalized with dispersed TGN38-positive structures, and some of the puncta were localized on discrete microdomains on plasma membrane (Extended Data Fig. 9a, upper left panel). This led us to speculate that PtdIns4P and TGN38 may be separated from each other after stimulation. This hypothesis is supported

by the fact that NLRP3(ΔKKKK/OSBP-PH)-GFP (which constitutively binds to PtdIns4P), although completely colocalized with TGN38 in a single cluster under basal conditions, formed distinct puncta that partially separated from TGN38-positive dispersed structures after imiquimod or CL097 treatment, and the puncta residing on plasma membrane usually lacked TGN38 signal (Extended Data Fig. 9a, lower left panel). This is in contrast to nigericin-induced NLRP3(ΔKKKK/OSBP-PH)-GFP puncta, which still localized on TGN38-positive vesicles (Extended Data Fig. 8b). It remains to be determined whether this unique feature of PtdIns4P separation from other TGN compartments contributes to the independence of NLRP3 activation on K^+ efflux by these stimuli. One possibility is the dramatic conformational change of PtdIns4P-enriched structures allows them to bind to the polybasic region of NLRP3 via ionic bonding even without the decrease in intracellular ionic strength caused by K^+ efflux. Nevertheless, NLRP3 activation induced by K^+ efflux-independent stimuli still requires its recruitment to PtdIns4P on dTGN in both reconstituted cells (Fig. 6a) and primary macrophages (Fig. 6d), again emphasizing the critical role of TGN dispersion and recruitment to dTGN in NLRP3 inflammasome activation by diverse stimuli.

Supplementary References

1. Tarantino, N. *et al.* TNF and IL-1 exhibit distinct ubiquitin requirements for inducing NEMO-IKK supramolecular structures. *J Cell Biol* **204**, 231-45 (2014).
2. Meng, G., Zhang, F., Fuss, I., Kitani, A. & Strober, W. A mutation in the Nlrp3 gene causing inflammasome hyperactivation potentiates Th17 cell-dominant immune responses. *Immunity* **30**, 860-74 (2009).
3. Brydges, S.D. *et al.* Inflammasome-mediated disease animal models reveal roles for innate but not adaptive immunity. *Immunity* **30**, 875-87 (2009).
4. Lu, A. *et al.* Unified polymerization mechanism for the assembly of ASC-dependent inflammasomes. *Cell* **156**, 1193-1206 (2014).

5. Cai, X. *et al.* Prion-like polymerization underlies signal transduction in antiviral immune defense and inflammasome activation. *Cell* **156**, 1207-1222 (2014).
6. Dick, M.S., Sborgi, L., Ruhl, S., Hiller, S. & Broz, P. ASC filament formation serves as a signal amplification mechanism for inflammasomes. *Nat Commun* **7**, 11929 (2016).
7. Proell, M., Gerlic, M., Mace, P.D., Reed, J.C. & Riedl, S.J. The CARD plays a critical role in ASC foci formation and inflammasome signalling. *Biochem J* **449**, 613-21 (2013).
8. Zhang, Z. *et al.* Protein kinase D at the Golgi controls NLRP3 inflammasome activation. *J Exp Med* **214**, 2671-2693 (2017).
9. Cheng, J. *et al.* Kinetic properties of ASC protein aggregation in epithelial cells. *J Cell Physiol* **222**, 738-47 (2010).
10. Hornung, V. *et al.* AIM2 recognizes cytosolic dsDNA and forms a caspase-1-activating inflammasome with ASC. *Nature* **458**, 514-8 (2009).
11. Burckstummer, T. *et al.* An orthogonal proteomic-genomic screen identifies AIM2 as a cytoplasmic DNA sensor for the inflammasome. *Nat Immunol* **10**, 266-72 (2009).
12. Szentpetery, Z., Varnai, P. & Balla, T. Acute manipulation of Golgi phosphoinositides to assess their importance in cellular trafficking and signaling. *Proc Natl Acad Sci U S A* **107**, 8225-30 (2010).
13. Hsu, F., Hu, F. & Mao, Y. Spatiotemporal control of phosphatidylinositol 4-phosphate by Sac2 regulates endocytic recycling. *J Cell Biol* **209**, 97-110 (2015).
14. Narayan, K. & Lemmon, M.A. Determining selectivity of phosphoinositide-binding domains. *Methods* **39**, 122-33 (2006).
15. Carlton, J.G. & Cullen, P.J. Coincidence detection in phosphoinositide signaling. *Trends Cell Biol* **15**, 540-7 (2005).
16. Balla, T. & Varnai, P. Visualizing cellular phosphoinositide pools with GFP-fused protein-modules. *Sci STKE* **2002**, pl3 (2002).
17. Traub, L.M., Ostrom, J.A. & Kornfeld, S. Biochemical dissection of AP-1 recruitment onto Golgi membranes. *J Cell Biol* **123**, 561-73 (1993).
18. Boman, A.L., Zhang, C., Zhu, X. & Kahn, R.A. A family of ADP-ribosylation factor effectors that can alter membrane transport through the trans-Golgi. *Mol Biol Cell* **11**, 1241-55 (2000).
19. Goud, B. & Gleeson, P.A. TGN golgins, Rabs and cytoskeleton: regulating the Golgi trafficking highways. *Trends Cell Biol* **20**, 329-36 (2010).
